# Cyclic AMP Receptor Protein Acts as a Transcription Regulator in Response to Stresses in *Deinococcus radiodurans*

**DOI:** 10.1371/journal.pone.0155010

**Published:** 2016-05-16

**Authors:** Su Yang, Hong Xu, Jiali Wang, Chengzhi Liu, Huizhi Lu, Mengjia Liu, Ye Zhao, Bing Tian, Liangyan Wang, Yuejin Hua

**Affiliations:** 1 Key Laboratory of Ministry of Agriculture for Nuclear-Agricultural Sciences, Institute of Nuclear-Agricultural Sciences, Zhejiang University, Hangzhou 310029, China; 2 Laboratory of Microbiology and Genomics, Zhejiang Institute of Microbiology, Hangzhou, China; Florida International University Bimolecular Sciences Institute, UNITED STATES

## Abstract

The cyclic AMP receptor protein family of transcription factors regulates various metabolic pathways in bacteria, and also play roles in response to environmental changes. Here, we identify four homologs of the CRP family in *Deinococcus radiodurans*, one of which tolerates extremely high levels of oxidative stress and DNA-damaging reagents. Transcriptional levels of CRP were increased under hydrogen peroxide (H_2_O_2_) treatment during the stationary growth phase, indicating that CRPs function in response to oxidative stress. By constructing all CRP single knockout mutants, we found that the *dr0997* mutant showed the lowest tolerance toward H_2_O_2_, ultraviolet radiation, ionizing radiation, and mitomycin C, while the phenotypes of the *dr2362*, *dr0834*, and *dr1646* mutants showed slight or no significant differences from those of the wild-type strain. Taking advantage of the conservation of the CRP-binding site in many bacteria, we found that transcription of 18 genes, including genes encoding chromosome-partitioning protein (*dr0998*), Lon proteases (*dr0349* and *dr1974*), NADH-quinone oxidoreductase (*dr1506*), thiosulfate sulfurtransferase (*dr2531*), the DNA repair protein UvsE *(dr1819*), PprA (*dra0346*), and RecN (*dr1447*), are directly regulated by DR0997. Quantitative real-time polymerase chain reaction (qRT-PCR) analyses showed that certain genes involved in anti-oxidative responses, DNA repair, and various cellular pathways are transcriptionally attenuated in the *dr0997* mutant. Interestingly, DR0997 also regulate the transcriptional levels of all CRP genes in this bacterium. These data suggest that DR0997 contributes to the extreme stress resistance of *D*. *radiodurans* via its regulatory role in multiple cellular pathways, such as anti-oxidation and DNA repair pathways.

## Introduction

Cyclic AMP (cAMP) receptor proteins (CRPs) are global transcriptional regulators that are widely distributed in bacteria[1, 2]. They play important roles in the regulation of many biological processes, including adaptation to starvation and extreme temperatures, energy metabolism, cell division, and toxin production[3–7]. As a transcription factor, the CRP/FNR family has diverse functions in different bacteria[8–11].

In *Escherichia coli*, CRP directly or indirectly controls the transcription of more than 400 genes, and it has been studied extensively[12, 13]. It regulates transcription through binding to the effector cAMP, which is synthesized by a membrane-bound adenylate cyclase (CyaA) in the absence of glucose[14]. Crystallographic studies of *E*. *coli* CRP have been performed to determine the structure of a CRP-cAMP complex, its target DNA, and the carboxyl-terminal domain of the RNA polymerase (RNAP) α-subunit[15–19]. It is shown that CRP contains a helix-turn-helix DNA-binding motif in its carboxyl-terminal domain and a cAMP-binding site in its amino-terminal domain[20–22]. Binding of cAMP causes a conformational change in CRP that leads to the formation of the CRP-cAMP complex[23–25], which can interact with an ~22-bp DNA-binding site with the consensus sequence 5′–AAATGTGATCTAGATCACATTT–3′[26]. However, the consensus DNA-binding sequences of CRP homologs from *Corynebacterium glutamicum* GlxR[27], *Mycobacterium tuberculosis* RV3676[28], and *Haemophilus influenza* CRP[29] differ slightly from that of *E*. *coli* CRP. Biochemical analyses reveal that CRP interacts with the carboxyl-terminal domain of the RNAP α-subunit[30]. This interaction facilitates binding of RNAP to the promoter, resulting in the initiation of transcription.

*Deinococcus radiodurans*, which belongs to the phylum *Deinococcus-Thermus*, is characterized by its extreme resistance to ionizing radiation, ultraviolet (UV) irradiation, desiccation, hydrogen peroxide (H_2_O_2_), and other DNA-damaging agents[31, 32]. Because of its efficient DNA repair ability and extreme stress resistance, *D*. *radiodurans* is generally considered to be an ideal model organism for studying bacterial resistance mechanisms under various stress conditions. There exists many predicted open reading frames (ORFs) encoding transcriptional factors in the *D*. *radiodurans* genome [33], whereas their functions, activities, and binding sites have rarely been elucidated.

*D*. *radiodurans* is predicted to contain four CRP family proteins, including DR0997, DR1646, DR2362, and DR0834. Interestingly, the transcriptional level of *dr0997*, also referred to as *ddrI* (DNA damage response gene I), increases 38-fold after ionizing radiation treatment[34], indicating that it might be involved in post-ionizing radiation recovery.

In this study, we constructed all the single mutants of CRPs and examined their roles in anti-oxidation, DNA repair, and other important cellular pathways. It was demonstrated that one of the CRP homologs, DR0997, acts as an important transcriptional activator that is involved in diverse cellular pathways, including cell growth, oxidative stress response, and DNA damage repair.

## Materials and Methods

### Bacterial strains, plasmids, media, and growth conditions

The *D*. *radiodurans* wild-type R1 (American Type Culture Collection 13939, Rockville, MD, USA), *E*. *coli* DH5α, and *E*. *coli* BL21 (DE3)pLysS strains were available in our laboratory. All *D*. *radiodurans* strains were grown at 30°C in tryptone-glucose-yeast extract (TGY) broth (0.5% tryptone, 0.1% glucose, 0.3% yeast extract) with aeration, or on TGY medium solidified with 1.5% w/v agar. When necessary, antibiotics were added to *D*. *radiodurans* cultures as follows: 30 μg mL^–1^ kanamycin, 10 μg mL^–1^ streptomycin, and 3.4 μg mL^–1^ chloramphenicol. All *E*. *coli* strains were grown at 37°C in Luria–Bertani medium (1% tryptone, 0.5% yeast extract, 1% NaCl) and supplemented with 30 μg mL^–1^ chloramphenicol, 50 μg mL^–1^ kanamycin, or 50 μg mL^–1^ ampicillin where appropriate. All strains and plasmids used in this study are listed in Table 1.

**Table 1:** Strains and plasmids used in this study.

Strains and plasmids	Description	Source
*D*. *radiodurans*		
R1	ATCC13939	[35]
*dr0997* mutant	*D*. *radiodurans dr0997* gene knockout mutant	This work
*dr2362* mutant	*D*. *radiodurans dr2362* gene knockout mutant	This work
*dr1646* mutant	*D*. *radiodurans dr1646* gene knockout mutant	This work
*dr0834* mutant	*D*. *radiodurans dr0834* gene knockout mutant	This work
*dr0997* mutant Cwt	*dr0997* mutant complement with pRAD*dr0997*	This work
*E*. *coli*		
DH5α	Host for cloning vectors	Takara
Bl21 (DE3) plysS	Host for expressing proteins	Takara
DR0997-HMT BL21	BL21 containing expression plasmid pET28a-HMT*dr0997*	This work
DR0615 BL21	BL21 containing expression plasmid pET28a-*dr0615*	This work
TEV-pET28a BL21	BL21 containing expression plasmid TEV-pET28a	[35]
Plasmids		
pET28a	Expression vector	Takara
pET28a-HMT	Expression vector reformed by pET28a with insertion of maltose affinity protein	[35]
pRADK	pRADZ3 derivative in which lacZ is replaced by the kanamycin gene	[36]
pRAD*dr0997*	pRADK derivative in which kanamycin gene is replaced by the *dr0997* gene	This work
pET28a-*dr0615*	pET28a expression plasmid containing BamHI-NdeI fragment of *dr0615*	This work
pET28a-HMT*dr0997*	pET28a-HMT expression plasmid containing BamHI-NdeI fragment of *dr0997*	This work

### CRP gene mutation and complementation in *D*. *radiodurans*

CRP null mutants, including *dr0997*, *dr2362*, *dr1646*, and *dr0834* null mutant, were constructed using the deletion replacement method as described previously[37]. Take the *dr0997* null mutant for example. Briefly, ~500-bp DNA fragments immediately upstream and downstream of *dr0997* were amplified from the genome of the wild-type R1 strain. After gel extraction, the two fragments were digested with BamHI and HindIII respectively, and ligated with a kanamycin resistance cassette fragment containing the *groEL* promoter, which was digested with the same enzymes. The kanamycin resistance cassette was obtained from pRADK, a shuttle plasmid derived from pRADZ3. The ligated product was then transformed into competent *D*. *radiodurans* cells in the exponential growth phase using CaCl_2_ as described previously[37]. The mutant strain was confirmed by genomic polymerase chain reaction (PCR) with the primers *dr0997*upF and *dr0997*downR (S1 Table). The full-length *dr0997* gene was amplified from the genome of the wild-type R1 strain with primers Com*dr0997*F and Com*dr0997*R (S1 Table) and digested with NdeI and BamHI. The predigested shuttle vector pRADZ3 was then ligated with the *dr0997* fragment and transformed into the *dr0997* null mutant to form the *dr0997*-complemented strain.

### Growth curves and survival tests

Growth curves of the CRP null mutant strains were determined by measuring the optical density at 600 nm (OD_600_). All strains were cultured in 5 ml of liquid TGY medium until the OD_600_ reached ~2.0, and then they were diluted 1:500 into new flasks containing 100 ml of TGY medium. The cultures were incubated at 30°C with shaking at 220 rpm, and samples were taken every 2 h to measure the OD_600_.

H_2_O_2_, UV, and mitomycin C (MMC) were used to test the mutants’ stress resistances. The survival rates of the CRP mutants after exposure to H_2_O_2_ were determined with a previously described method [38]. Briefly, cells were diluted to an appropriate concentration with 10 mM MgSO_4_. H_2_O_2_ (Sigma-Aldrich, St. Louis, MO, USA) was added to the cell suspension to a final concentration of 50 mM, and the cells were incubated at room temperature. Samples were obtained at 0, 5, 10, 20, and 40 min, and catalase (30 mg/ml) (Sigma-Aldrich) was added to inactive the H_2_O_2_. The survival fraction (%) was calculated using the following equation: survival fraction (%) = N_sample_/N_control_ × 100%, where N_control_ is the number of wild-type bacteria and N_sample_ is the number of mutant bacteria. For the UV sensitivity test, cultures with an OD_600_ of ~1.0 were diluted with 10 mM MgSO_4_. After plating onto TGY agar plates, the plates were exposed to different dosages of UV radiation by calculating the exposure time as described previously[38]. For the MMC sensitivity assay, cells were diluted with 10 mM MgSO4 and treated with a final concentration of 5 μg/ml MMC (Sigma-Aldrich), and then plated onto TGY agar plates every 10 min. All the plates were incubated at 30°C for 3 d. All data shown are the means ± SDs of three independent experiments.

### Measurement of catalase and total antioxidant capacity

Cells were cultured at 30°C in 100 ml of TGY medium for at least 12 h until the OD_600_ reached ~1.0. Half of the culture was treated with 30 mM H_2_O_2_ for 30 min, harvested, washed twice with phosphate buffer (20 mM, pH 7.4), and suspended in 2 ml of phosphate buffer.

Catalase activity was determined as described previously[39]. The collected cells were disrupted on ice with an ultrasonic cell disruptor at an output of 450 W for 15 min. Debris was removed by centrifugation, and the concentration of soluble protein was determined using the Bradford method. The soluble supernatant was diluted with an appropriate chromogenic reagent mixture (Beyotime, China). The quantity of H_2_O_2_ remaining in the mixture is determined by the oxidative production of N-(4-antipyryl)-3-chloro-5-sulfonate-p-benzoquinonemonoimine in the presence of H_2_O_2_ when catalyzed by horseradish peroxidase in the chromogenic reagent mixture. After a 15-min incubation, the resultant product was quantified at 520 nm.

The total antioxidant capacity was assayed as reported previously[40]. Briefly, 2, 2′-azino-bis (3-ethylbenzthiazoline-6-sulfonic acid (ABTS)) was used as a dye solution. An appropriately diluted protein solution was mixed with the oxidant regent for 6 min, and the generation of ABTS^.+^ was measured at 734 nm. The total antioxidant capacity was presented by comparing it with the antioxidant activity of Trolox (Beyotime).

### RNA extraction and quantitative real-time polymerase chain reaction (qRT-PCR) assay

Total RNA extraction and data processing were conducted as described previously[41]. Briefly, the wild-type strain and *dr0997* mutant strain were cultured until the OD_600_ reached 0.4, and then harvested. Total RNA was extracted using the Whole RNA Extraction kit (Promega, Madison, WI USA). RNA quality and quantity were evaluated by measuring the A_260_/A_280_ ratio with a NanoDrop-1000 spectrophotometer (NanoDrop Technologies Inc., Wilmington, DE, USA) and Denaturing Agarose Gel Electrophoresis.

The qRT-PCR assay utilized RNA samples that obtained under different conditions. The wild-type and mutant strains were grown in TGY until the OD_600_ reached 0.5. Each culture was divided in two halves: one half of the culture was treated with H_2_O_2_ at a final concentration of 50 mM, while the other half was used as the non-treated control. Total RNA was extracted as mentioned previously. First-strand cDNA synthesis was conducted in 20-μl reactions containing 1 μg of purified RNA and 3 mg of random hexamers. The SYBR Green PCR kit (Tiangen, Beijing, China) was used for PCR amplification according the manufacturer’s instructions, and all assays were performed using the Mx3005P^TM^ Real-time Detection System (Stratagene, La Jolla, CA, USA).

### Protein expression and purification

Proteins were induced and purified as described previously[42, 43]. Briefly, the *dr0997* fragment was obtained by digesting pRAD-*dr0997* with NdeI and BamHI, and it was ligated with pET28a-HMT. pET28a-HMT is a pET-28a derivative that encodes maltose binding protein (MBP) containing specific tobacco etch virus (TEV) protease recognition sites. *E*. *coli* BL21 (DE3) pLysS cells carrying plasmid pET28a-HMT*dr0997* were grown to an OD_600_ of 0.6 at 37°C and induced with 200 μM isopropyl β-D-1-thiogalactopyranoside at 37°C for at least 12 h. Cells were harvested by centrifugation, washed twice with phosphate buffer (20 mM, pH 7.4), and resuspended in binding buffer (500 mM NaCl, 20 mM Tris, 5% glycerol, pH 8.0). Cells were disrupted on ice with an ultrasonic cell disruptor at an output of 350 W for 20 min. After centrifugation at 15,000 × *g* for 20 min at 4°C, the supernatant was loaded onto a HisTrap HP column (GE Healthcare, Little Chalfont, UK). The purified DR0997-MBP fusion protein was digested with a proper concentration of TEV protease at 4°C overnight, and then loaded onto a maltose-affinity column. The eluted protein was applied to a HiTrap Q HP column to remove undigested protein and imidazole. The purity of the protein sample was determined using 12% sodium dodecyl sulfate-polyacrylamide gel electrophoresis (SDS-PAGE), and only the fraction containing pure DR0997 protein was used for further experiments.

### Electrophoretic mobility-shift assay (EMSA)

EMSA was performed with DNA fragments (100 ng) mixed with 2 μM purified DR0997 or DR0615 protein in a total volume of 10 μl. The binding buffer contained 10 mM Tris-HCl (pH7.5), 10 mM KCl, 200 mM NaCl, 5 mM MgCl_2_, 5 mM MnCl_2_, 10 μg mL^–1^ bovine serum albumin, 200 μM cAMP, and 100 μM dithiothreitol[44]. The reaction mixture was incubated at 30°C for 30 min and then loaded onto 12% (w/v) polyacrylamide gels in 1×Tris-borate-ethylenediamine tetraacetic acid buffer. Electrophoresis was performed at 100 V for 6 h at 4°C, and the gel was stained with ethidium bromide and photographed (Typhoon 9500, GE Healthcare). The *E*. *coli* CRP-binding site was used to identify predicted CRP-regulated genes in *D*. *radiodurans*. The promoter of *dr1998* was used as a positive control of DR0615. The *dr0167* promoter and coding region of *dr0167* (*dr0167*C) were used as negative controls. All primers are listed in S1 File.

## Results

### Characterization of *D*. *radiodurans* CRPs

**Fig 1:**
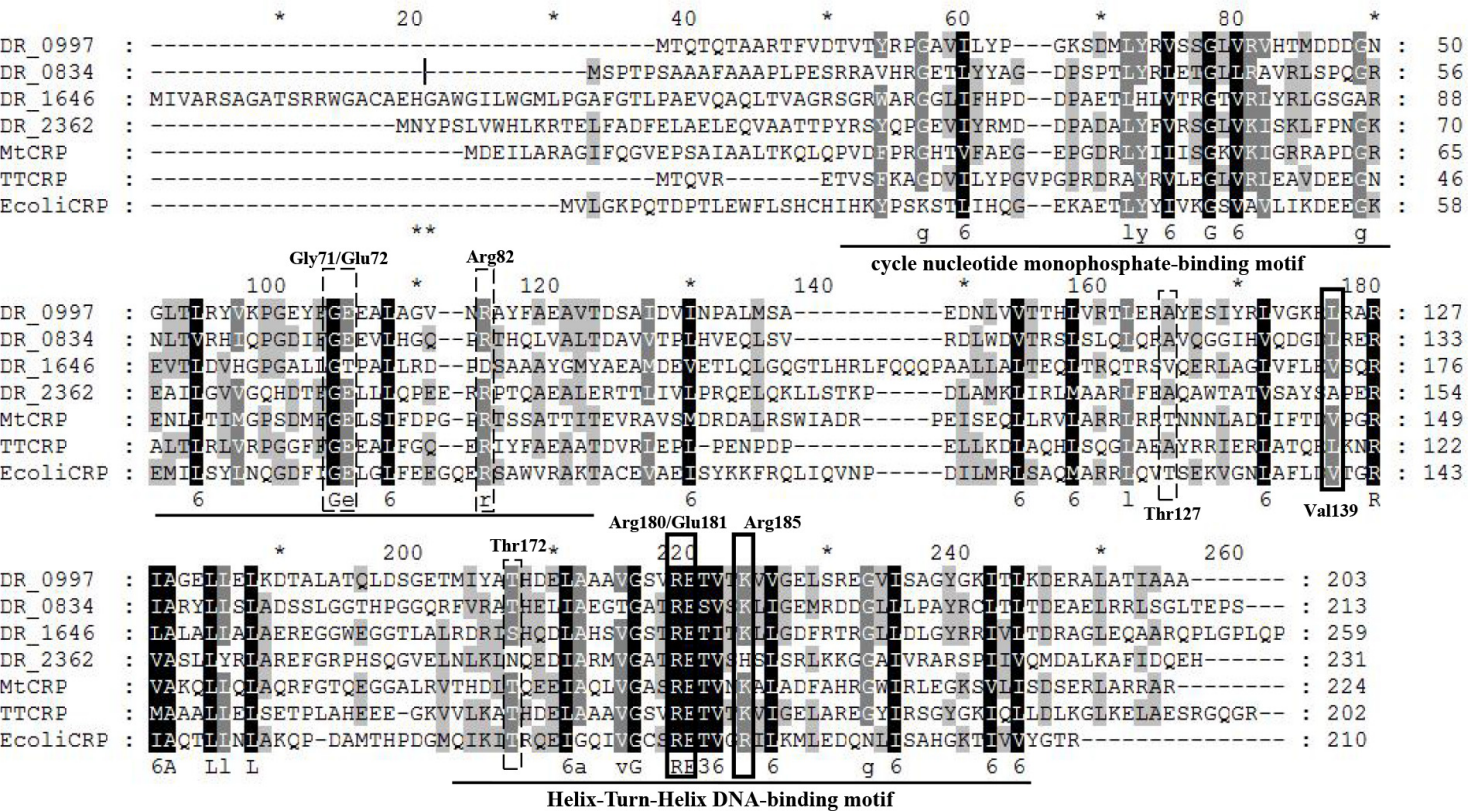
Alignment of CRP homologs from different organisms. ClustalX software was used to align the amino acid sequence of *D*. *radiodurans* R1 DR0997, DR0834, DR1646, and DR2362, *M*. *tuberculosis* RV3676, *T*. *thermophilus* HB8 TTHA1357, and *E*. *coli* CRP. The conservative residues Gly71, Glu72, Arg82 and Thr172 (dotted frames) of *E*. *coli* CRP are required for cAMP binding, while Arg180 and Glu181 (solid frames) are required for DNA binding, based on the crystal structure of *E*. *coli* CRP.

The *dr0997* ORF is predicted to encode a 260-amino acid protein (National Center for Biotechnology Information accession no. AAF10573.1). However, based on our sequencing results, a base was missing at the 543-bp site, resulting in the shortening of the *dr0997* ORF (S1 Fig). The corrected DR0997, DR2362, DR0834, and DR1646 exhibit nearly 23% identity to *E*. *coli* CRP, as indicated by a protein Basic Local Alignment Search Tool (BLASTP) analysis. The conserved helix-turn-helix and the nucleotide monophosphate-binding regions are underlined in Fig 1. Based on the crystal structure of CRP-cAMP, CRP (cAMP)-DNA and CRP-CTD-DNA complex., the residues required for cAMP binding were marked with dotted frame, while the residues required for DNA binding were marked with solid frame It is observed that 4 residues (Gly71, Glu72, Arg82 and Thr172) required for cAMP binding and 2 residues (Arg180 and Glu181) required for DNA binding are conserved in *E*. *coli* CRP [45–49]. Unlike most of the CRP/FNR family proteins, there is no cysteine residue in *D*. *radiodurans* CRP that could sense oxygen or redox variations [50–52].

**Fig 2:**
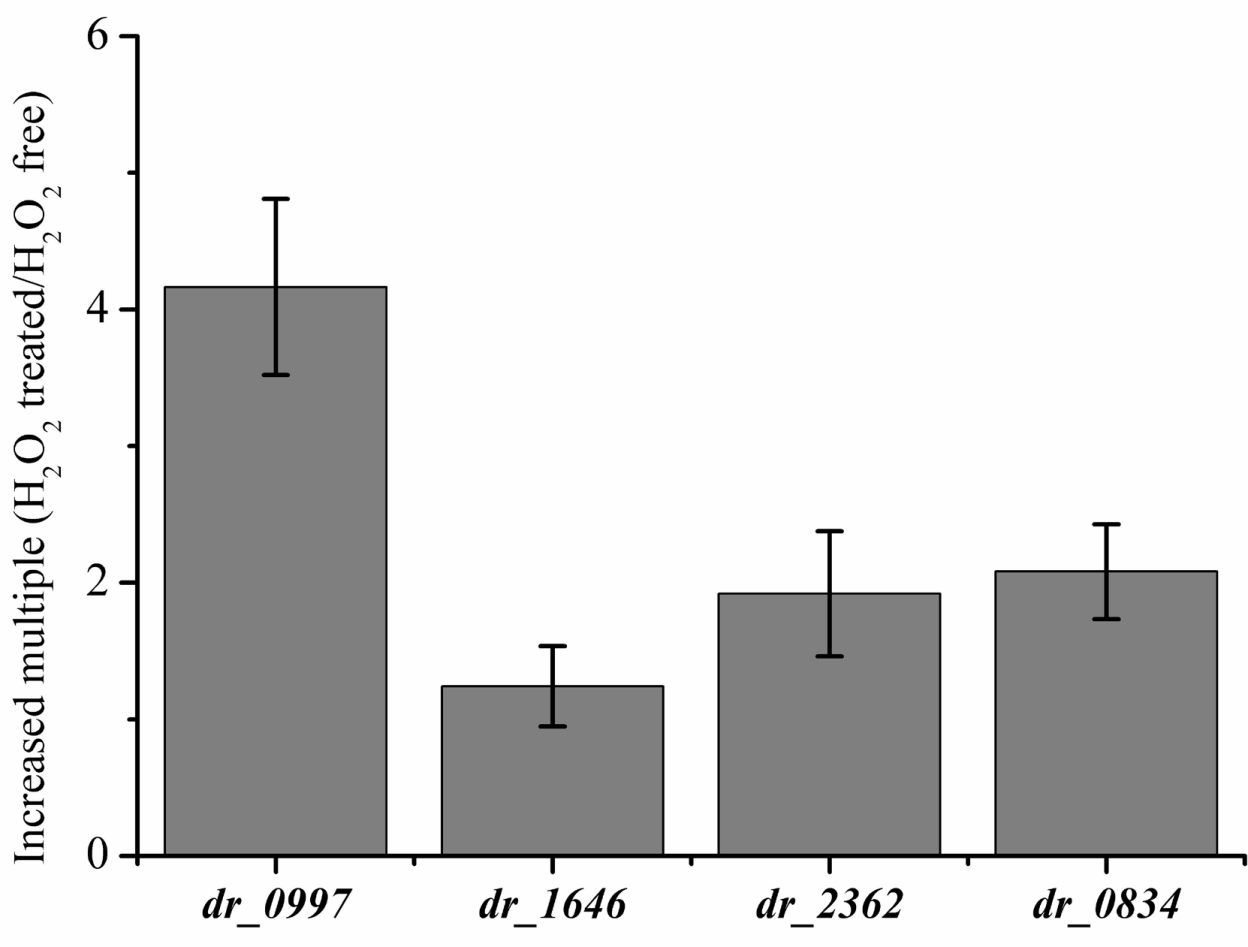
Increased multiple of transcriptional levels of CRP genes in *D*. *radiodurans* R1 after exposure to H_2_O_2_ compared with the untreated samples. Data represent the averages and standard deviations of three independent experiments.

The alteration of expression of CRP genes in *D*. *radiodurans* in response to H_2_O_2_ treatment were detected compared to the untreated sample, and statistical analyses were applied for data processing (S1 Table). It was demonstrated that the expression levels of CRP genes are increased to different extents when the bacteria were exposed to oxidizing agents. Specifically, the expression of *dr0997 i*s induced 4-fold, indicating that it might be a crucial player in the oxidative stress response (Fig 2). Meanwhile, the expression of *dr0997* increases greatly following post-irradiation recovery [34]. These results demonstrate the important roles of CPRs in stress resistance in *D*. *radiodurans*.

### The *dr0997* mutant exhibits a growth defect

**Fig 3:**
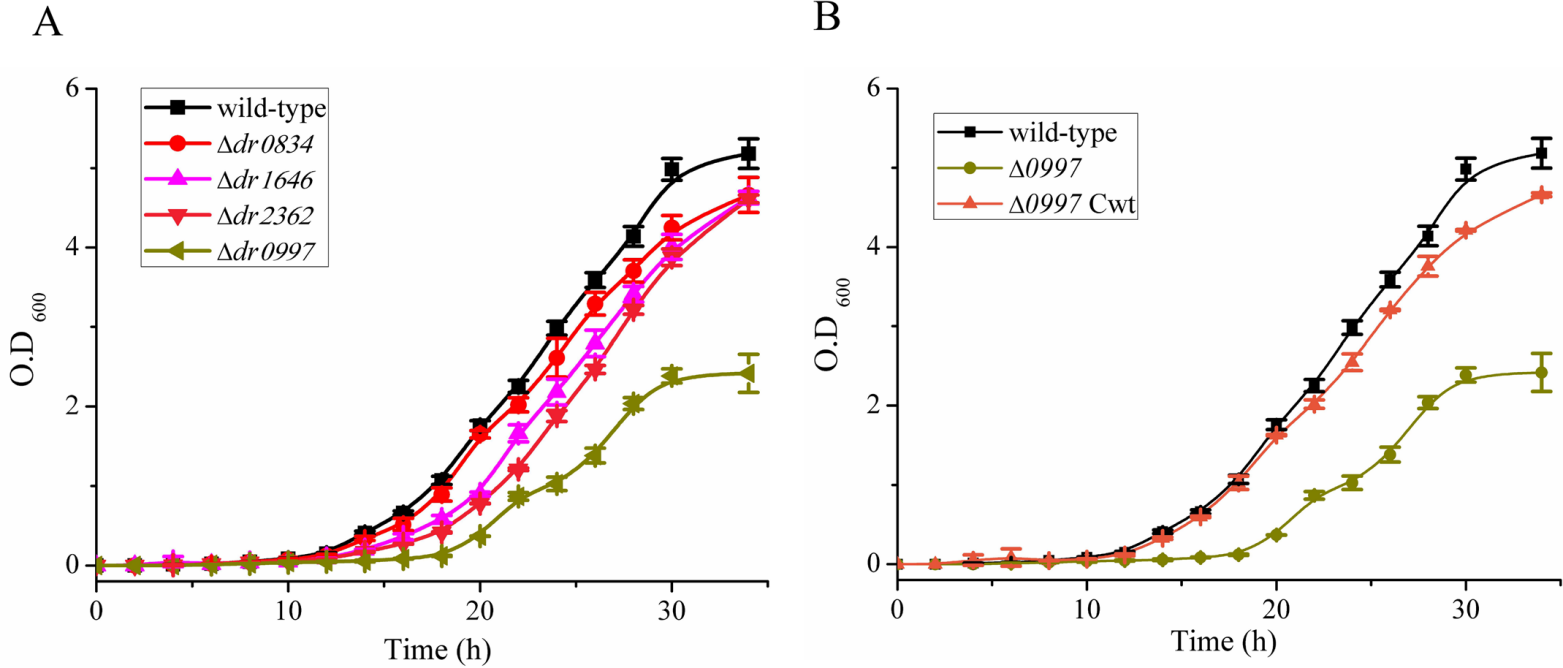
Growth curves of *D*. *radiodurans* strains. (A) Wild-type and four CRP mutants were individually grown in TGY medium, and the OD_600_ was measured every 2 h. (B) Growth rates of the wild-type, *dr0997* mutant, and the *dr0997* mutant complemented with the *dr0997* gene (*dr0997 mutant* Cwt) were recorded every 2 h.

Through a double crossover recombination strategy, four CRP mutants were generated by replacing the CRP-encoding genes with a kanamycin cassette (S2 Fig). After growth in TGY broth without antibiotics, bacterial growth rates were monitored using a spectrophotometer at 2-h internals. As shown in Fig 3, only the *dr0997* mutant exhibited an obvious growth defect, and the wild-type growth rate was restored in the *dr0997*-complemented strain. The *dr2362*, *dr0834*, and *dr1646* mutants only showed slight growth defects. These data indicated that *dr0997* is necessary for normal growth.

### Deletion of *dr0997* results in sensitivity to various stresses

**Fig 4:**
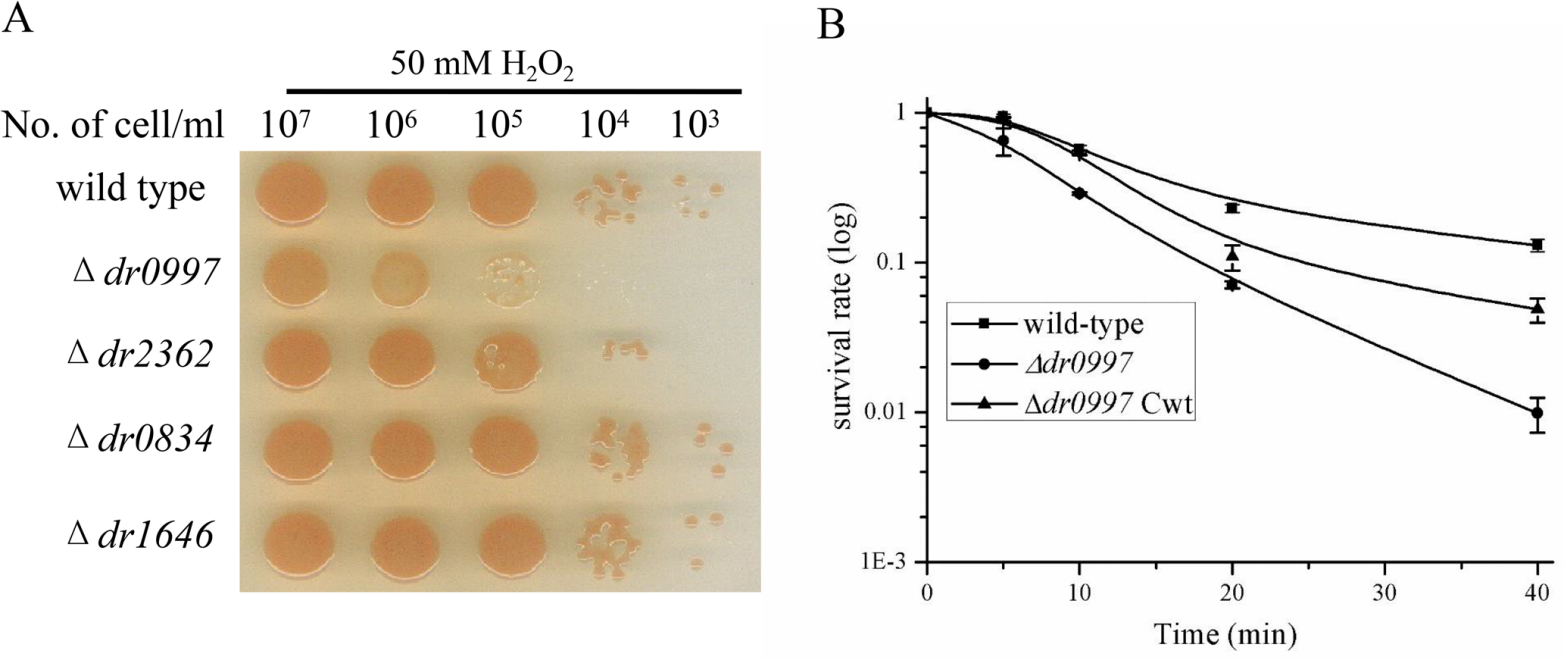
Survival curves of *D*. *radiodurans* strains exposed to 50 mM H_2_O_2_. (A) Wild-type and CRP mutants exposed to 50 mM H_2_O_2_ for 20 min, and then plated on TGY agar plates. (B) Wild-type, *dr0997* mutant, and the *dr0997* mutant complemented with the *dr0997* gene (*dr0997 mutant* Cwt) were exposed to 50 mM H_2_O_2_ for different periods of time. Data represent the averages and standard deviations of three independent experiments.

To identify the role of *D*. *radiodurans* CRPs in stress resistances, the survival of the CRP mutants were measured and the statistical analyses were applied for data processing (S2 Table). The results showed that the *dr0997* mutant was more susceptible to H_2_O_2_ than the wild-type strain, as it exhibited a more than 10-fold decrease in survival after treatment with 50 mM H_2_O_2_ for 20 min (Fig 4A). The H_2_O_2_ resistance of the mutant recovered partly when complemented with the *dr0997* gene (pRAD0997) (Fig 4B), and the statistical analysis also indicated the complemented action has statistical significance, suggesting that *dr0997* is responsible for the oxidative resistance of the bacterium. However, the *dr2362* mutant was only slightly sensitive to oxidative stress (S3 Fig and S3 Table), while the *dr0834* and *dr1646* mutants were not sensitive to oxidative stress (Data not shown).

**Fig 5:**
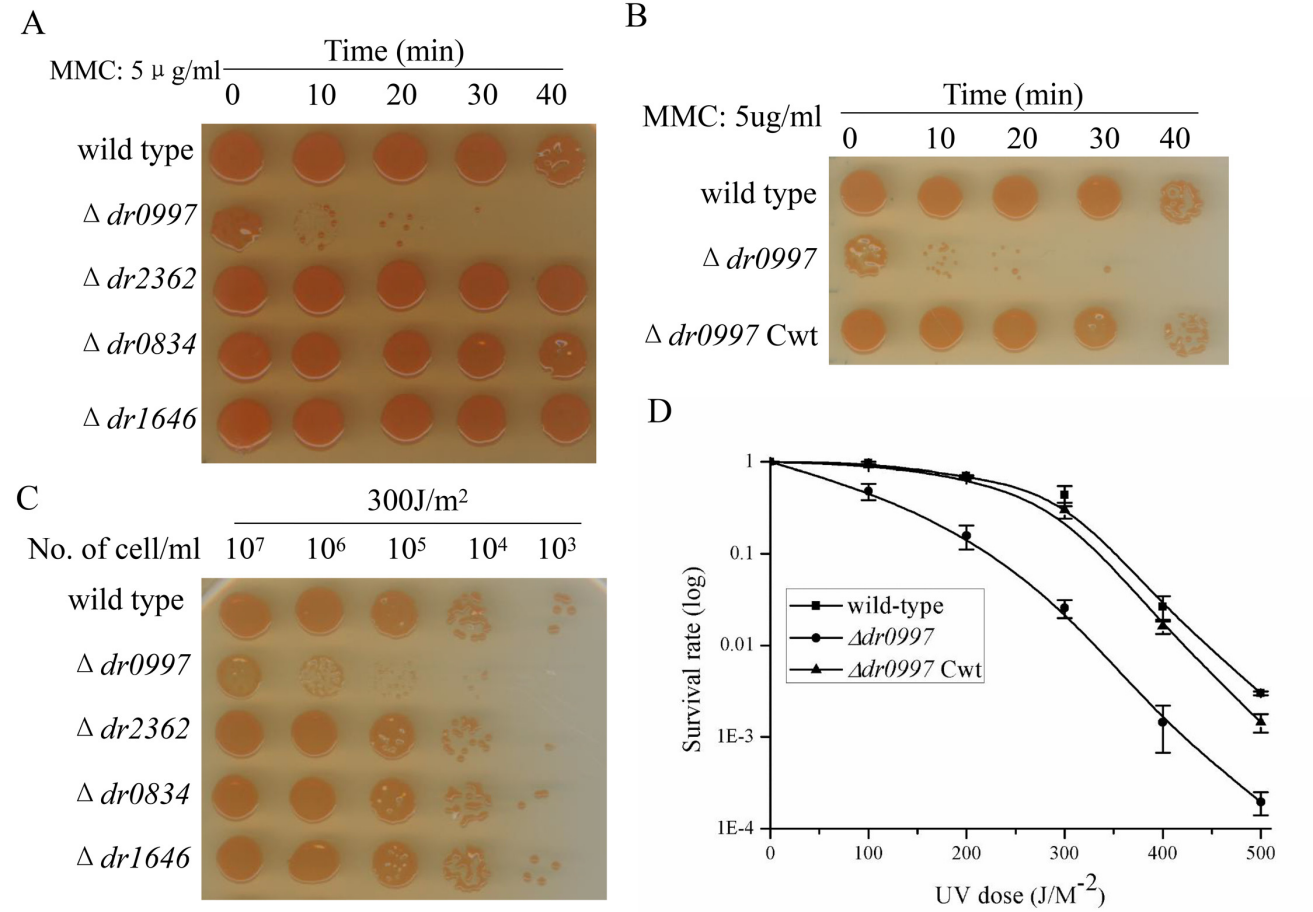
Survival of CRP mutants exposed to MMC and UV radiation. (A) Survival of CRP mutants and the wild-type strain after exposure to 5 μg/ml MMC for different periods of time. (B) Survival of the *dr0997* mutant and the *dr0997* mutant complemented with the *dr0997* gene (*dr0997 mutant* Cwt) after MMC exposure. (C) Survival of the CRP mutants and the wild-type strain after exposure to UV radiation. (D) Survival curves of the wild-type, *dr0997* mutant, and *dr0997 mutant* Cwt strains after exposure to UV radiation (0 to 500 Jm^−2^). Data represent the averages and standard deviations of three independent experiments.

**Fig 6:**
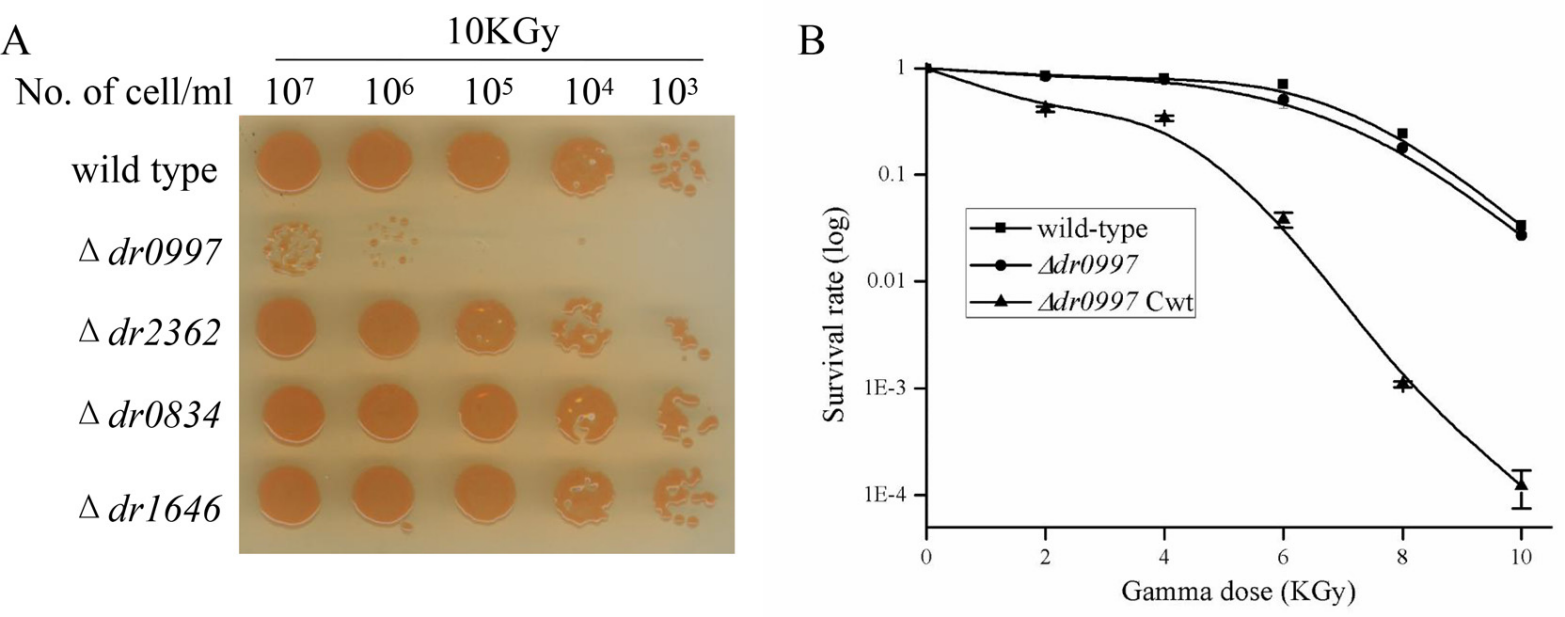
Survival of CRP mutants after exposure to gamma radiation. (A) Survival of CRP mutants and the wild-type strain after exposure to 10 kGy of gamma radiation. (B) Survival curves of the *dr0997* mutant and the *dr0997* mutant complemented with the *dr0997* gene (*dr0997 mutant* Cwt) after exposure gamma radiation. Data represent the averages and standard deviations of three independent experiments.

Similar results were obtained in response to other stresses, such as UV radiation, ionizing radiation, and MMC. Disruption of *dr0997* resulted in a dramatic increase in sensitivity to UV, gamma radiation, and MMC, which was restored by complementation with the *dr0997* gene (Figs 5 and 6). Either 200 J m^−2^ of UV radiation or 5 kGy of gamma ray radiation were sufficient to kill 95% of the *dr0997* mutant cells. Meanwhile, the *dr0997* mutant only survived 5 μg/ml MMC for 20 min, while the other CRP mutants did not shown any survival differences at this dosage. These results reveal the key role of *dr0997* in stress resistances.

### Disruption of *dr0997* lowers anti-oxidation activity

**Fig 7:**
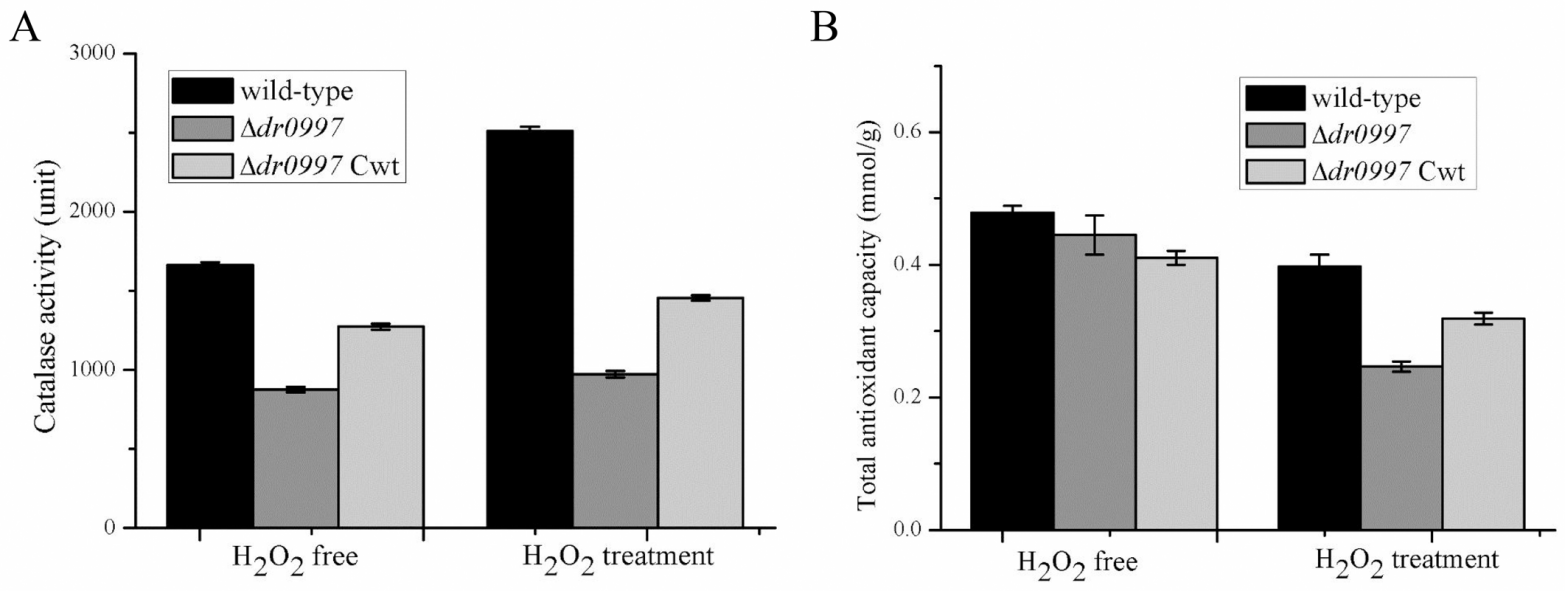
Effect of *dr0997* disruption on the anti-oxidant ability of *D*. *radiodurans*. (A) Total antioxidant capacity of the wild-type R1, *dr0997* mutant, and *dr0997* mutant complemented with the *dr0997* gene (*dr0997 mutant* Cwt) strains after exposure to 30 mM H_2_O_2_ treatment. (B) Catalase activities after 30 mM H_2_O_2_ treatment. Data represent the average and standard deviations of three independent experiments.

The *dr0997* mutant of *D*. *radiodurans* displayed decreased H_2_O_2_ resistance, suggesting that DR0997 may be involved in the cellular anti-oxidative response. Under normal conditions, as shown in Fig 7A, the *dr0997* mutant did not show any significant decrease in total anti-oxidant capacity. However, when treated with 30 mM H_2_O_2_ for 30 min, the anti-oxidative activity of the *dr0997* mutant decreased by nearly 50%, compared with a 20% decrease in the wild-type and *dr0997*-complemented strains. These data are consistent with the H_2_O_2_ survival phenotype of the *dr0997* mutant.

Catalase activity was measured under normal growth conditions or in response to H_2_O_2_ treatment. The results showed that the *dr0997* mutant exhibited less catalase activity than the wild-type strain under normal conditions (Fig 7B). When treated with 30 mM H_2_O_2_ for 30 min, the wild-type strain showed a significant increase (approximately 40%) in catalase activity. However, catalase activity was barely induced in the *dr0997* mutant, whereas the catalase activity in the *dr0997*-complemented strain was nearly equal to that of the wild-type strain, indicating that cells lacking the *dr0997* gene lose the ability to cope with oxidative stress. The data were subjected to statistical analysis, and the differences were statistically significant (S4 Table).

### DR0997 regulates numerous genes via a specific CRP-binding site

CRP has been shown to be a global transcriptional regulator that is involved in many cellular pathways, such as carbon utilization, lycopene synthesis, antibiotic production, virulence factor regulation, and anti-oxidative stress responses[3, 53–55]. Based on the phenotype of the *dr0997* mutant in response to oxidative stress, DNA damage, and other stresses, we focused on the characterization of *dr0997* in anti-oxidative and DNA damage response pathways.

**Fig 8:**
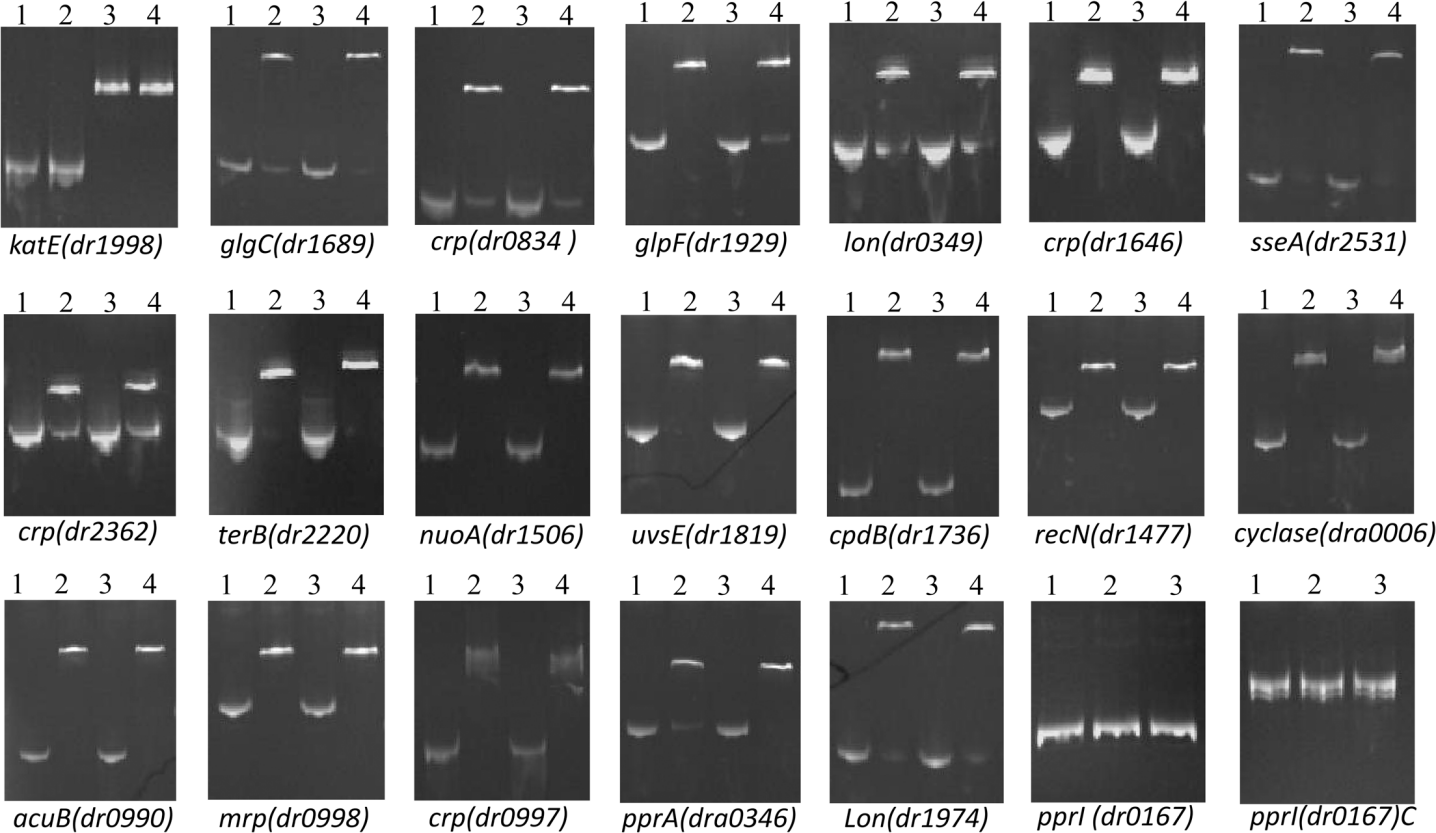
Electrophoretic mobility shift assays of DR0997 binding to the upstream regions of different genes with predicted CRP binding sites. Lane 1, 100 ng of the indicated DNA fragment; lane 2, 100 ng of the indicated DNA fragment with the DR0997 protein. Lane 3, 100 ng of the indicated DNA fragment with the DR0615 protein. Lane 4, 100 ng of the indicated DNA fragment with the DR0997 and DR0615 proteins. The promoter of *dr1998* was used as a positive control of DR0615. The *dr0167* promoter and coding region of *dr0167* (*dr0167*C) were used as negative controls.

**Fig 9:**
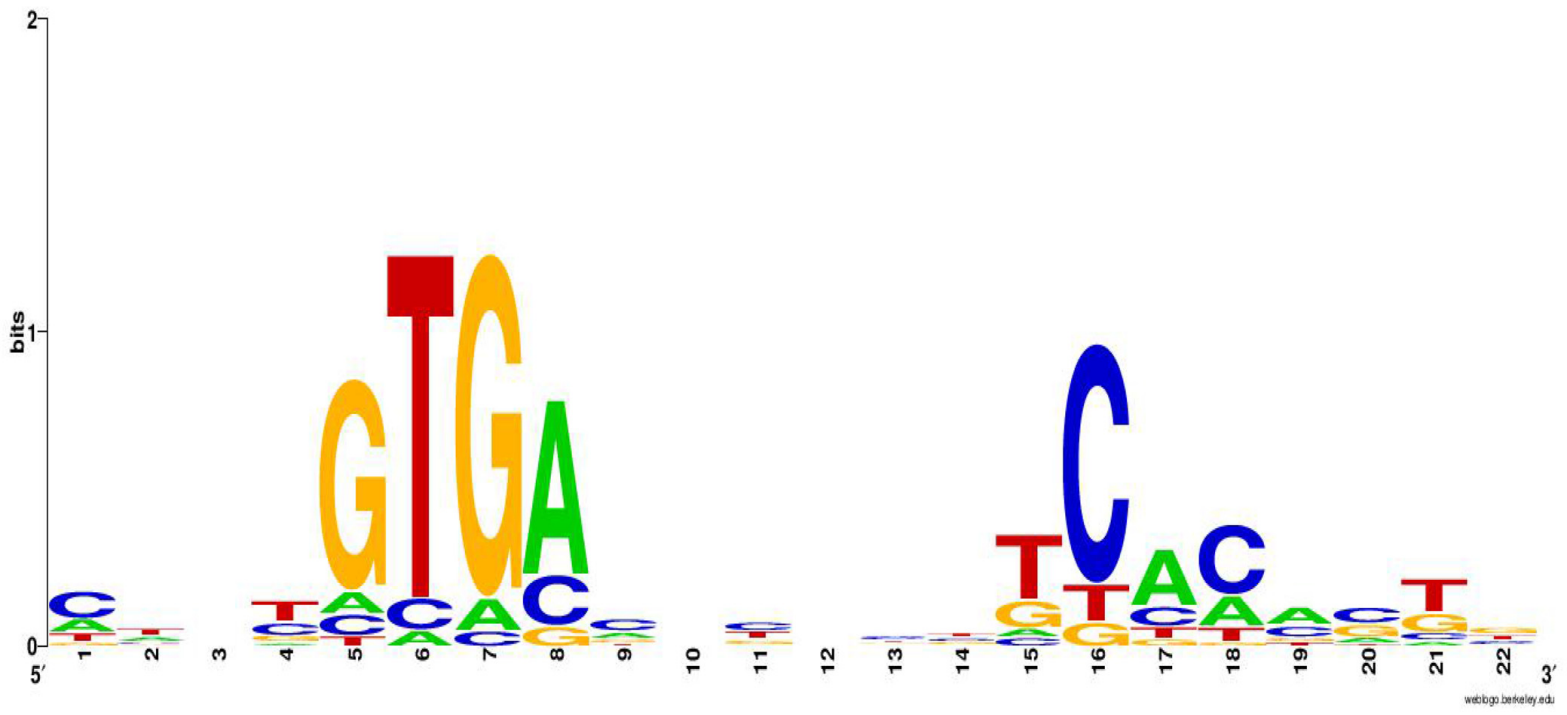
Sequence logo of the predicted CRP-binding sites in *D*. *radiodurans*. The height of each stack of letters represents the degree of sequence conservation measured in bits. The height of each letter within a stack is proportional to its frequency at that position in the binding site. The letters are sorted with the most frequent on top. This sequence logo was generated using the online WebLogo (Univ. of California at Berkeley, Berkeley, CA, USA) program (http://weblogo.berkeley.edu/logo.cgi).

By virtue of the consensus CRP-binding site in *E*. *coli*, promoters of numerous anti-oxidation or DNA repair related genes were checked to see whether they contain similar CRP binding sites. Meawhile, the homologues which has been already proved to be regulated by CRP in other bacteria were also analysed[3, 52, 56, 57]. Finally, 18 genes containing CRP-binding sites in their promoters were shown to be directly regulated by DR0997 via EMSAs (Fig 8). Interestingly, most of these promoters contain at least two similar CRP-binding sites. To verify the precise binding site, the regions containing two or more predicted CRP binding sites were segmented into two or more DNA frgments with their own primers respectively, and re-tested by EMSA. It was demonstrated that in most of promoters including *dr1506*, *dr1477*, *dr0998*, *dra0006*, *dr0990*, and *dr1689*, both segments could be bound by DR0997. However, in other promoters including *dr1819*, *dr1974*, *dr1929*, *dr1736*, only one segment would interact with DR0997. The confirmed CRP-binding sites (S4 Fig) are listed in S2 File. Based on the confirmed binding sites, the CRP-binding consensus sequence in *D*. *radiodurans* was predicted in Fig 9. Classification of the genes that are directly regulated by DR0997 revealed that nine genes are involved in stress resistance, and eight genes are involved in metabolic pathways.

### Induction of anti-oxidation-related genes is attenuated in the *dr0997* mutant

As a predicted global transcription regulator, DR0997 may control a series of genes that is directly or indirectly involved in oxidative resistance. Using reverse transcription-PCR method, the transcriptional levels of a series of anti-oxidative enzymes, including *katE* (*dr1998* and *dra0259*), *katA* (*dra0146*), *sodC* (*dr1546* and *dra0202*), *sodA* (*dr1279*), *terB* (*dr2220*), and NADH dehydrogenase (*dr1506*) were measured. As shown in Table 2, prior to H_2_O_2_ treatment, the transcriptional levels of *katE* (*dr1998* and *dra0259*), *katA* (*dra0146*), and *sodC* (*dra0202*) were 2-fold lower in the mutant than in the wild-type strain. After treatment with 50 mM H_2_O_2_, the transcriptional levels in the mutant were still lower than those of the wild-type strain, which is in accordance with the aforementioned catalase activity.

**Table 2:** qRT PCR of anti-oxidation-related genes in the *dr0997* mutant relative to wild-type R1 with or without H_2_O_2_ treatment.

Locus	Annotation	Fold change (±SD)	
		H_2_O_2_ free	H_2_O_2_ treatment
		Δ0997(-H)/R1(-H)	Δ0997(+H)/R1(+H)
DR1998	Catalase (KatE)	-5.47 (±1.20)	-2.30 (±0.30)
DRA0259	Catalase (KatE)	-2.04 (±0.37)	-1.09 (±0.11)
DRA0146	Catalase (CatA)	-2.00 (±0.24)	-1.33 (±0.20)
DR1546	Cu/Zn family superoxide dismutase (SodC)	0.86 (±0.02)	0.81 (±0.03)
DRA0202	Cu/Zn family superoxide dismutase (SodC)	-3.41 (±0.65)	-1.26 (±0.07)
DR1279	Mn family superoxide dismutase (SodA)	-1.27 (±0.03)	-1.34 (±0.09)
DR2220	Tellurite resistance protein TerB	0.74 (±0.22)	-3.52 (±0.65)
DR1506	NADH-quinone oxidoreductase subunit A	0.76 (±0.14)	-1.38 (±0.24)

Functional annotation is based on KEGG (http://www.genome.jp/kegg/).

Specifically, the *dr1506* and *dr2220* genes were directly regulated by DR0997. DR1506 is an NADH-quinone oxidoreductase that is involved in redox maintenance. The DR2220 (TerB) knockout strain exhibited reduced H_2_O_2_ resistance compared with the wild-type strain (S5 Fig). Combined with the aforementioned survival results, we propose that certain antioxidant enzymes, such as catalase or anti-oxidation-related proteins like TerB and NADH dehydrogenase, are dependent on the expression of DR0997, suggesting that DR9997 might act as a positive regulator under oxidative stress.

### Induction of DNA repair-related genes is attenuated in the *dr0997* mutant

To reveal how the *dr0997* deletion affects the bacterial survival rate in response to DNA-damaging agents_,_ such as UV radiation, gamma radiation, and MMC, the transcriptional levels of an array of DNA damage response and DNA repair genes were measured by qRT-PCR under normal conditions and in response to gamma radiation treatment. Twenty-five genes were selected for the qRT-PCR analysis, including *recA*, *recN*, *recG*, *recF*, *recJ*, *recO*, *uvrB*, *uvrC*, *uvsE*, *ddrO*, *priA*, *drRRA*, *ddrB*, *ddrC*, and *ddrD* (Table 3).

**Table 3:** qRT PCR of DNA repair related genes in the *dr0997* mutant relative to wild-type R1 with or without gamma treatment.

Locus	Annotation	Fold change (±SD)	
		γ free	γ treatment
		Δ0997(-γ)/R1(-γ)	Δ0997(+γ)/R1(+γ)
DR1477	DNA repair protein RecN	-3.46 (±0.32)	-2.38 (±0.03)
DRA0346	DNA damage repair protein PprA	-2.79 (±0.29)	-1.77 (±0.19)
DR1819	putative UV damage endonuclease UvsE	-5.19 (±0.45)	-1.36 (±0.17)
DR1771	excinuclease ABC subunit A UvrA	-1.11 (±0.30)	0.81 (±0.06)
DR2275	excinuclease ABC subunit B UvrB	-3.21 (±0.39)	-1.87 (±0.21)
DR1354	excinuclease ABC subunit C UvrC	-5.95 (±0.53)	-2.99 (±0.31)
DR1274	Holliday junction DNA helicase RuvA	-1.13 (±0.02)	-0.93 (±0.13)
DR0596	Holliday junction DNA helicase RuvB	-1.09 (±0.06)	0.98 (±0.09)
DR0440	crossover junction endodeoxyribonuclease RuvC	-7.54 (±0.98)	-4.57 (±0.83)
DR0400	cell division protein FtsK	-1.36 (±0.12)	-1.35 (±0.09)
DR1916	ATP-dependent DNA helicase RecG	-2.68 (±0.16)	-2.78 (±0.09)
DR2340	recombination protein RecA	-15.56 (±0.75)	-8.27 (±0.39)
DR2606	primosome assembly protein PriA	-2.58 (±0.38)	-2.57 (±0.11)
DR2418	DNA-binding response regulator DrRRA	-1.61 (±0.04)	-1.81(±0.02)
DR0167	Radiation response protein PprI	-1.50 (±0.63)	-1.05 (±0.02)
DR0003	tellurite resistance protein TerB	-14.59 (±1.32)	-11.08 (±1.24)
DR0070	radiation induced single-stranded DdrB	-6.35 (±0.27)	-2.06 (±0.14)
DR1440	cation-transporting ATPase	0.86 (±0.07)	-5.68 (±0.35)
DR2574	XRE family transcriptional regulator DdrO	-3.82 (±0.25)	-1.00 (±0.03)
DR0326	radiation-induced protein	-7.13 (±0.65)	-2.19 (±0.14)
DR1126	single-stranded-DNA-specific exonuclease	-2.02 (±0.16)	-3.25 (±0.56)
DR1089	DNA replication and repair protein RecF	-4.81 (±0.15)	-2.11 (±0.03)
DR0819	DNA replication and repair protein RecO	-2.64 (±0.05)	0.94 (±0.01)
DR0198	DNA replication and repair protein RecR	0.78 (±0.06)	0.76 (±0.02)

Functional annotation is based on KEGG (http://www.genome.jp/kegg/).

The expression of 17 of the 25 genes was significantly down- or up-regulated in the *dr0097* mutant, suggesting that these genes are transcriptionally affected by DR0997. Under normal conditions, the transcriptional levels of *recA*, *priA*, and *recG* were 23-, 10-, and 4-fold lower, respectively, in the *dr0997* mutant than those of the wild type strain. RuvABC is required for the formation of Holliday junctions, as well as for recombination[58]. UvrABC has been reported to be involved in DNA excision repair[59, 60]. The transcriptional levels of these genes were all lower in the *dr0997* mutant. The expression of a newly identified repressor of DNA damage response gene, *ddrO*[61], was 3.8-fold lower in the *dr0997* mutant. DdrO and PprI mediate a novel DNA damage response pathway that differs from the classic LexA-mediated SOS response system in the radiation-sensitive bacterium *E*. *coli* [41]. The expression of several other DNA damage response proteins, including *ddrB*, *ddrC* and *ddrD*, was also 2.5-fold lower in the *dr0997* mutant. Although their precise functions remain to be illustrated, their important roles in the DNA damage response have already been confirmed [62].

Additionally, the transcriptional level of *drRRA* was nearly 2.9-fold lower in the *dr0997* mutant than in the wild-type strain, indicating that DR0997 might be involved in the feedback regulation of *drRRA* expression [38]. The transcriptional levels of most of the above genes were also lower in the *dr0997* mutant strain under ionizing radiation treatment (Table 3).

Among the detected genes, *uvsE* (*dr1819*), *pprA* (*dra0346*), and *recN* (*dr1477*) were shown to be directly regulated by DR0997 (Fig 8). RecN mainly functions in response to double-stranded DNA breaks, and it acts as a cohesion-like protein that stimulates intermolecular DNA interactions [63, 64]. PprA is regarded as a RecA-independent, DNA repair-related protein in *D*. *radiodurans*[62, 65, 66]. UvsE encodes a UV damage endonuclease that is involved in nucleotide excision repair[67]. From the results of the qRT-PCR analysis, the transcript levels of these genes were lower in the *dr0997* mutant than in the wild-type strain under normal conditions and in response to ionizing radiation treatment, indicating that they play crucial roles in DNA repair.

### Genes in other cellular pathways are also affected in the dr0997 mutant

As previously mentioned, CRP is a global transcriptional regulator that is involved in many cellular pathways [3, 53–55]. Based on the EMSA results, the genes that are directly regulated by DR0997 can be classified into diverse cellular pathways, including oxidative response and DNA repair pathways. The transcriptional levels of these genes were also measured under normal conditions or ionizing radiation treatment (Table 4).

**Table 4:** qRT PCR of directly regulated genes in the *dr0997* mutant relative to wild-type R1.

Locus	Annotation	Fold change (±SD)	
		γ free	γ treatment
		Δ0997(-γ)/R1(-γ)	Δ0997(+γ)/R1(+γ)
DR0997	CRP/FNR family transcriptional regulator	0.12 (±0.01)	0.17 (±0.01)
DR0834	CRP/FNR family transcriptional regulator	-1.03 (±0.08)	-1.21 (±0.07)
DR1646	CRP/FNR family transcriptional regulator	-1.56 (±0.11)	-2.35 (±0.12)
DR2362	CRP/FNR family transcriptional regulator	-2.78 (±0.09)	-1.95 (±0.12)
DR0990	acetoin utilization protein AcuB	0.77 (±0.09)	-2.00 (±0.29)
DR0998	ATP-binding protein involved in chromosome partitioning(mrp)	-3.10 (±0.11)	-2.72 (±0.04)
DR1736	2',3'-cyclic-nucleotide 2'-phosphodiesterase 3'-nucleotidase CpdB	-1.13 (±0.15)	-1.47 (±0.19)
DR1974	ATP-dependent protease LA	-2.71 (±0.22)	-5.48 (±1.06)
DRA0006	cyclase/dehydrase	-3.42 (±0.13)	-1.40 (±0.15)
DR1929	glycerol uptake facilitator protein GlpF	-1.14 (±0.07)	-4.07 (±0.13)
DR1689	glucose-1-phosphate adenylyltransferase GlgC	-22.02 (±1.04)	-24.25 (±0.20)
DR2531	thiosulfate sulfurtransferase	-2.02 (±0.19)	-2.92 (±0.02)
DR0349	ATP-dependent protease LA	-3.78 (±0.13)	-5.54 (±0.93)

Functional annotation is based on KEGG (http://www.genome.jp/kegg/).

Of the identified genes, *glpF* (*dr1929*) and *glgC* (*dr1689*) are involved in glycometabolism. *Dr0349* and *dr1974* encode proteins that belong to the Lon protease family, which can degrade and recycle damaged proteins, suggesting that DR0997 participates in protein degradation. *CpdB* (*dr1736*) encodes a cAMP phosphodiesterase that catalyzes the conversion of cAMP to AMP, while *dra0006* encodes a CyaA-like cyclase, indicating that DR0997 might regulate cAMP metabolism. *Dr0998* encodes an ATP-binding protein that is involved in chromosome partitioning, which implies that it functions in chromosome partitioning during cell division. Meanwhile, the *dr2531* gene encodes a thiosulfate sulfurtransferase that was also regulated by DR0997. Because the oxidation of thiols would result in non-native disulfide bond formation in proteins[52] and cause protein damage, it is suggested that DR0997 may have a role in regulating sulfur transfers and the prevention of thiol oxidation.

Furthermore, it is interesting that DR0997 could regulate not only its own expression, but also that of the other CRP homologs in *D*. *radiodurans*, indicating that DR0997 can regulate its own expression to cope with changes in the environment.

## Discussion

The regulation of gene expression is critical for a cell’s response and survival in various environments. As global transcriptional factors, CRPs have been investigated in diverse bacteria, and they are considered to be important players in the responses to various environmental changes. In this study, an analysis of survival of CRP knockout strains, as well as the transcriptional responses of CRP-regulated genes, revealed that one of the CRPs (DR0997) plays vital roles in *D*. *radiodurans*.

The first CRP regulation model was in *E*. *col* several decades ago[68]. Just like *E*. *coli* CRP, *D*. *radiodurans* CRPs contain a cyclic nucleotide-binding domain in their amino-terminus, as well as a helix-turn-helix domain that bindings DNA and recruits RNAP to promoters to activate transcription. The crystal structure of an *E*. *coli* CRP-DNA complex showed that the CRP dimer binds to the TGTGA and TCACA blocks of the 22-bp consensus CRP binding site. The consensus CRP-binding sites in other CRP homologs, such as *C*. *glutamicum* GlxR, *M*. *tuberculosis* Rv3676, and *Thermus thermophilus* HB8 TTHA1437, are highly similar. Using WebLogo, we obtained the sequence logo of DR0997-binding sites (TGTGA-N6-TCACA) based on the aforementioned EMSA data (Fig 9), which is somewhat similar to the consensus binding site of *E*. *coli* CRP (AAATGTGA-N6-TCACATTT) and other CRP family proteins[52, 69]. The specific binding site should be defined by further biochemical experiments. In *E*. *coli* CRP, cAMP acts as an essential signaling molecule, and it helps to form the CRP-DNA complex. FNR uses the redox states of bound metals as signals to regulate the expression of other genes[70] The *M*. *tuberculosis* genome encodes 15 adenylate cyclases, showing the importance of cAMP[71]. However, *D*. *radiodurans* does not contain a classical adenylate cyclase that catalyzes the synthesis of cAMP. Nevertheless, an alternation of the cAMP concentration after radiation has been reported[72], indicating that some uncharacterized proteins encode adenylate cyclases. DR0997 was also observed to regulate the expression of *dra0006*, which contains domains that are similar to those of adenylate cyclases, and *dr1736* (*cpdB)*, which catalyzes the conversion of cAMP to AMP, implying a connection between DR0997-mediated gene regulation and cAMP metabolism.

*E*. *coli* CRP has been demonstrated to play its anti-oxidation role by regulating the expression of *rpoS*, which activates the general stress response[73]. Using an error-prone PCR technique, CRP was engineered to improve oxidative stress resistance[55]. The *T*. *thermophilus* HB8 CRP/FNR family protein SdrP is regarded as an oxidative stress-responsive transcriptional activator[74], and it was shown to control the promoters of *sodA* and a thioredoxin reductase-encoding gene, which are known to participate in redox control and oxidative resistance. However, there are no *rpoS* homologs in *D*. *radiodurans*, and the CRP family protein DR0997 does not bind the promoters of *sodA* (*dr1279*) and *katE* (data not shown). Nevertheless, it was demonstrated that DR0997 binds the promoters of *dr1506*, which encodes NADH-quinone oxidoreductase subunit A, and *terB* (*dr2220*). DR1506 is involved in redox control, while DR2220 was reported to respond to oxidative stress[75]. These data suggest that DR0997 plays a role in transcriptional regulation during anti-oxidant processes.

For decades, CRPs have been regarded to be involved in metabolism and anti-oxidation pathways. However, our investigations revealed that DR0997 also directly regulates the expression of DNA repair-related genes, including *recN*, *pprA*, and *uvsE*. Many DNA damage response and repair genes were indirectly regulated by DR0997. It will be interesting to further clarify the function of DR0997 in DNA damage responses and DNA repair.

Beyond the impact of DR0997 on cells via its regulatory role in oxidation and DNA repair responses, its ability to regulate other cellular pathways could also affect cell survival. The regulation of expression of Lon protease and thiosulfate sulfurtransferase would directly affect cell viability in response to damage, while regulating glycometabolism would affect energy production. The regulation of the cAMP pathway also affects many cell activities. Further studies of DR0997 will contribute to a detailed understanding of its functions on the regulatory network during stress responses.

## Supporting Information

S1 Fig The corrected open reading frame of *dr0997*. (TIF)

S2 Fig Identification of CRP mutant strains. **(A)** All of the mutants were identified using their upF and downR (S1 File) primers, respectively. Lane 1, the wild-type strain; lane 2, the indicated mutant respectively. (B) The complemented strain and the mutant strain were identified using com0997F and com0997R primers, respectively. Lane 1, the *dr0997* mutant complemented with *dr0997* gene; Lane 2, the Δ*dr0997* strain.
(TIF)

S3 Fig Survival curves of Δ*2362* and wild-type strains exposed to 50 mM H_2_O_2_. Wild-type and *dr2362* mutant were exposed to 50 mM H_2_O_2_ for different periods of time. Data represent the averages and standard deviations of three independent experiments.
(TIF)

S4 Fig Electrophoretic mobility shift assays of DR0997 binding to the upstream regions of *dr1477*. Lane 1, 100 ng of the indicated DNA fragment; lanes 2 to 6, 100 ng of the indicated DNA fragment with increasing concentrations of DR0997 (0.1, 0.25, 0.5, 1, and 10 μM, respectively).
(TIF)

S5 Fig Survival curves of Δ*dr2220* strain exposed to 40 mM H_2_O_2_. (TIF)

S6 Fig Purification of DR0997. Lane 1, protein ladder; lane 2, DR0997 protein.
(TIF)

S7 Fig Purification of DR0615. Lane 1, protein ladder; lane 2, DR0615 protein.
(TIF)

S8 Fig Electrophoretic mobility shift assays of DR0997 binding to the upstream regions of *dr1477*. Lane 1, 100 ng of the indicated DNA fragment; lanes 2 to 6, 100 ng of the indicated DNA fragment with increasing concentrations of DR0997 (0.1, 0.25, 0.5, 1, and 10 μM, respectively).
(TIF)

S1 File Primers used in this study. (DOC)

S2 File CRP-binding sites. (DOC)

S1 Table Statistical analysis of increased multiple transcript of CRP genes after exposure to H_2_O_2_ (DOCX)

S2 Table Statistical analysis of survival curves of *D*. *radiodurans* strains exposure to 50 mM H_2_O_2_. (DOCX)

S3 Table Statistical analysis of survival curves of Δ*2362* and wild-type strains exposure to 50 mM H_2_O_2_. (DOCX)

S4 Table Statistical analysis of Catalase activities after 30 mM H_2_O_2_ treatment. (DOCX)
