# Supplementary figures and images for: Cyclic AMP Receptor Protein Acts as a Transcription Regulator in Response to Stresses in Deinococcus radiodurans
Source: PLoS One. 2016 May 16;11(5):e0155010. doi: 10.1371/journal.pone.0155010 (PMC4868304; doi:10.1371/journal.pone.0155010)

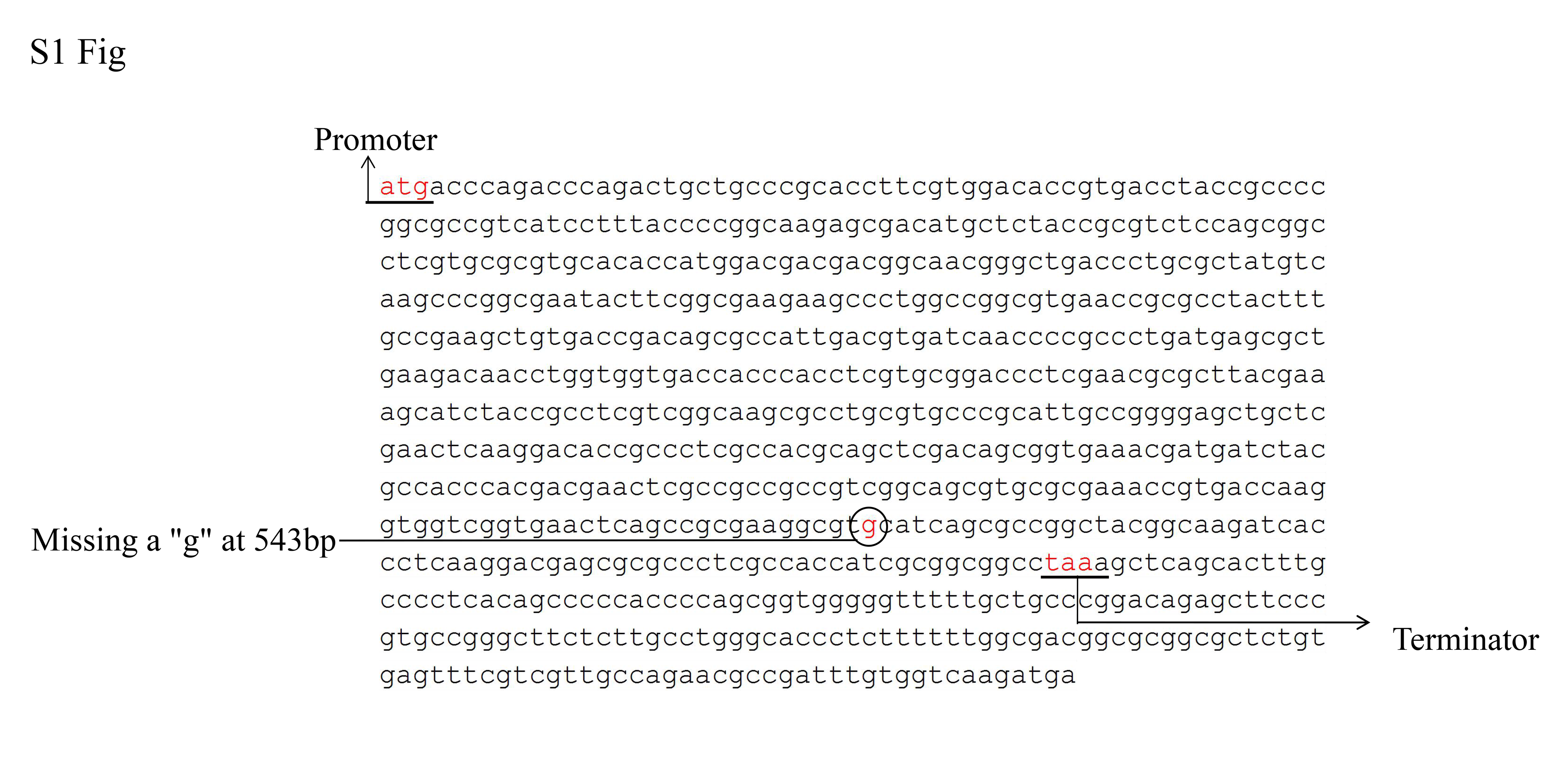

Supplement: S1 Fig — (TIF) [file pone.0155010.s001.tif]

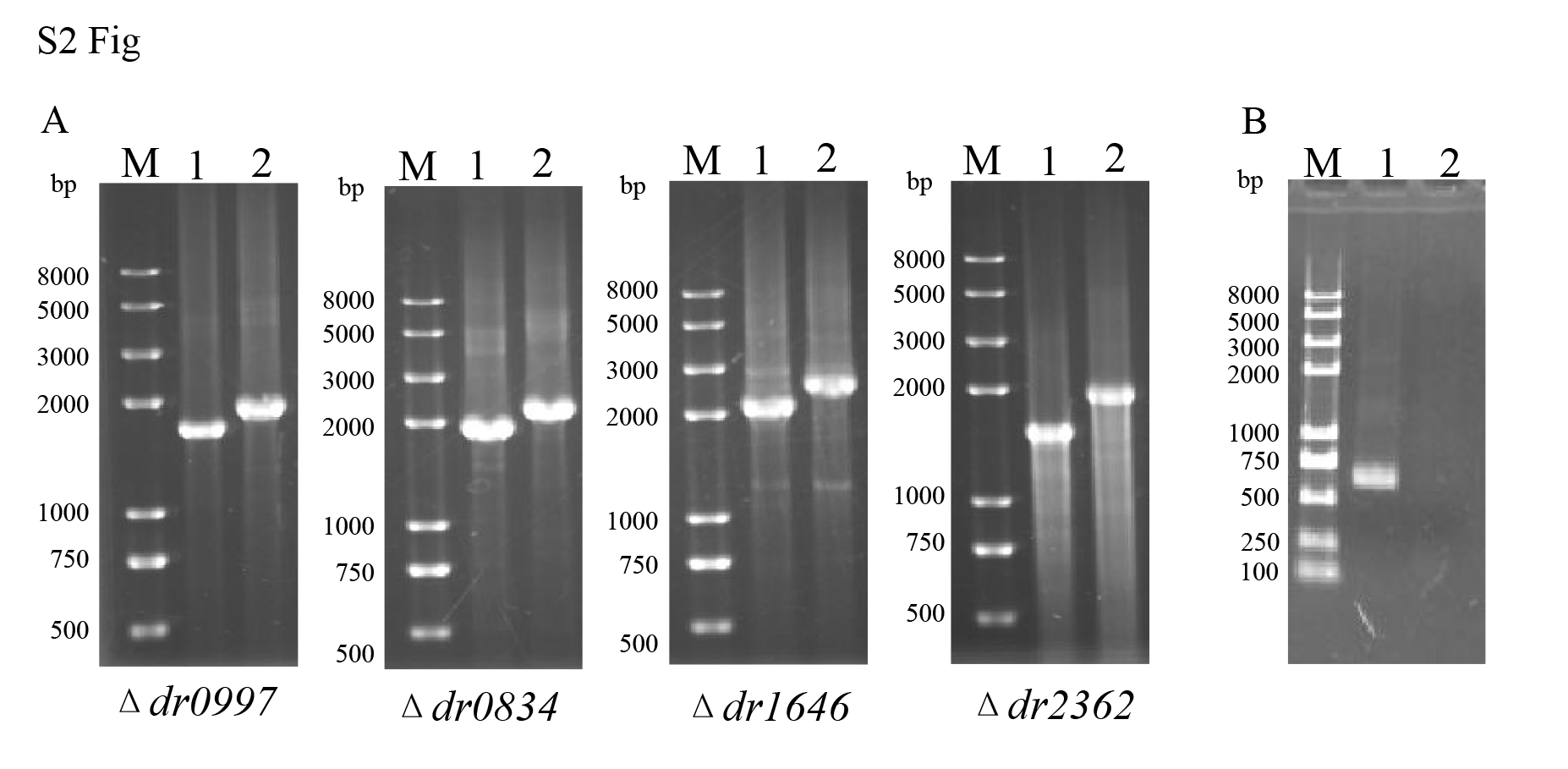

Supplement: S2 Fig — (A) All of the mutants were identified using their upF and downR (S1 File) primers, respectively. Lane 1, the wild-type strain; lane 2, the indicated mutant respectively. (B) The complemented strain and the mutant strain were identified using com0997F and com0997R primers, respectively. Lane 1, the dr0997 mutant complemented with dr0997 gene; Lane 2, the Δdr0997 strain. (TIF) [file pone.0155010.s002.tif]

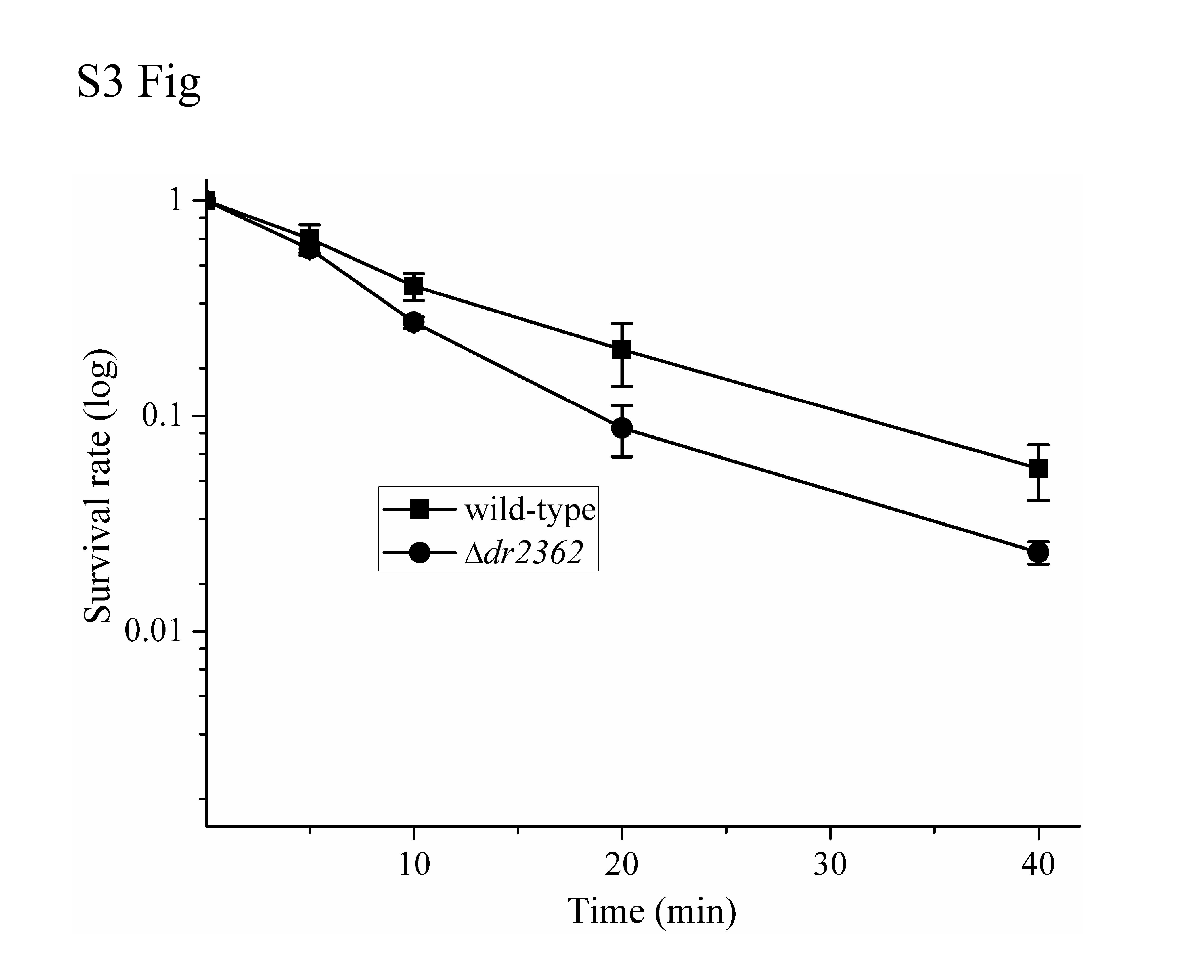

Supplement: S3 Fig — Wild-type and dr2362 mutant were exposed to 50 mM H2O2 for different periods of time. Data represent the averages and standard deviations of three independent experiments. (TIF) [file pone.0155010.s003.tif]

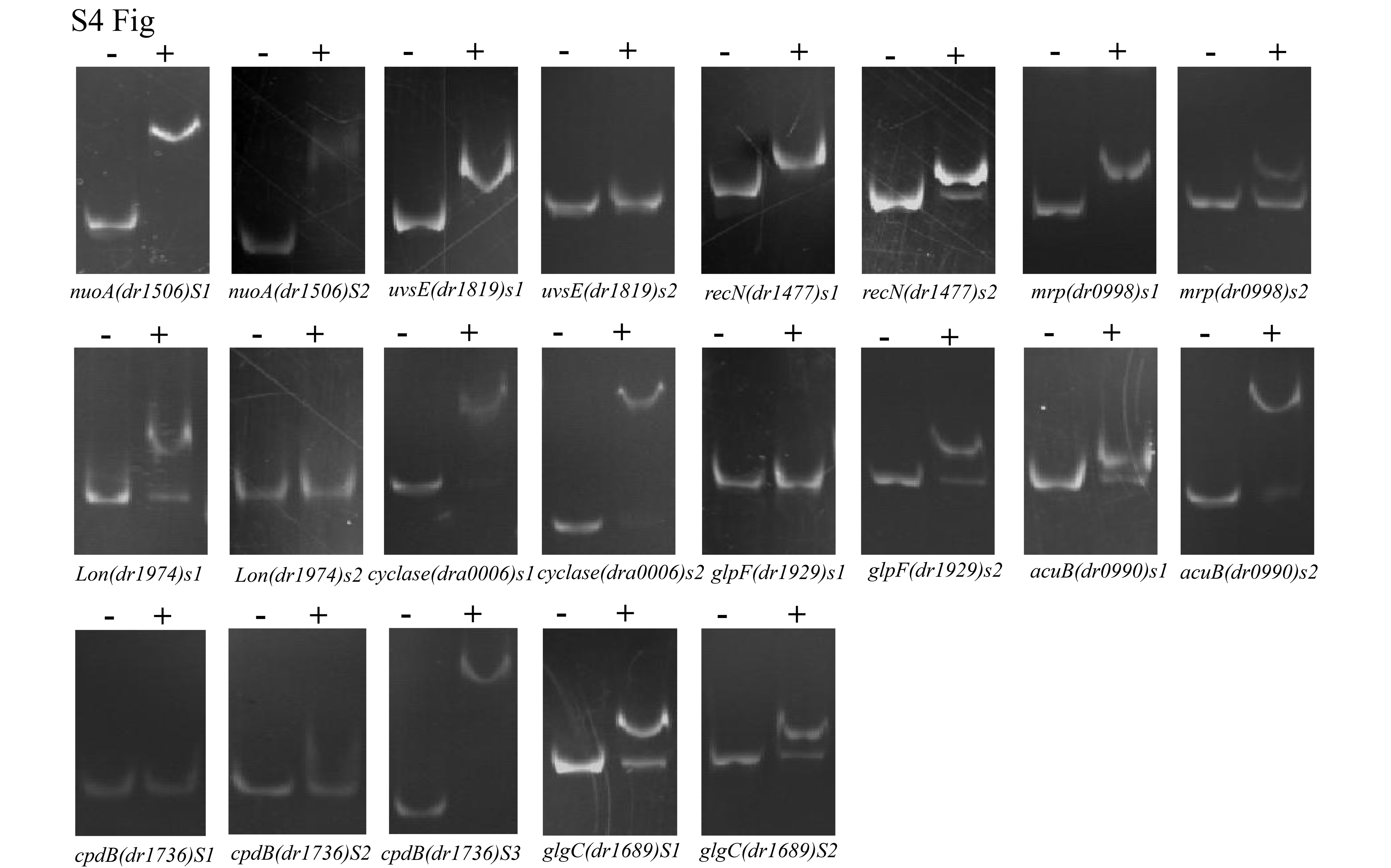

Supplement: S4 Fig — Lane 1, 100 ng of the indicated DNA fragment; lanes 2 to 6, 100 ng of the indicated DNA fragment with increasing concentrations of DR0997 (0.1, 0.25, 0.5, 1, and 10 μM, respectively). (TIF) [file pone.0155010.s004.tif]

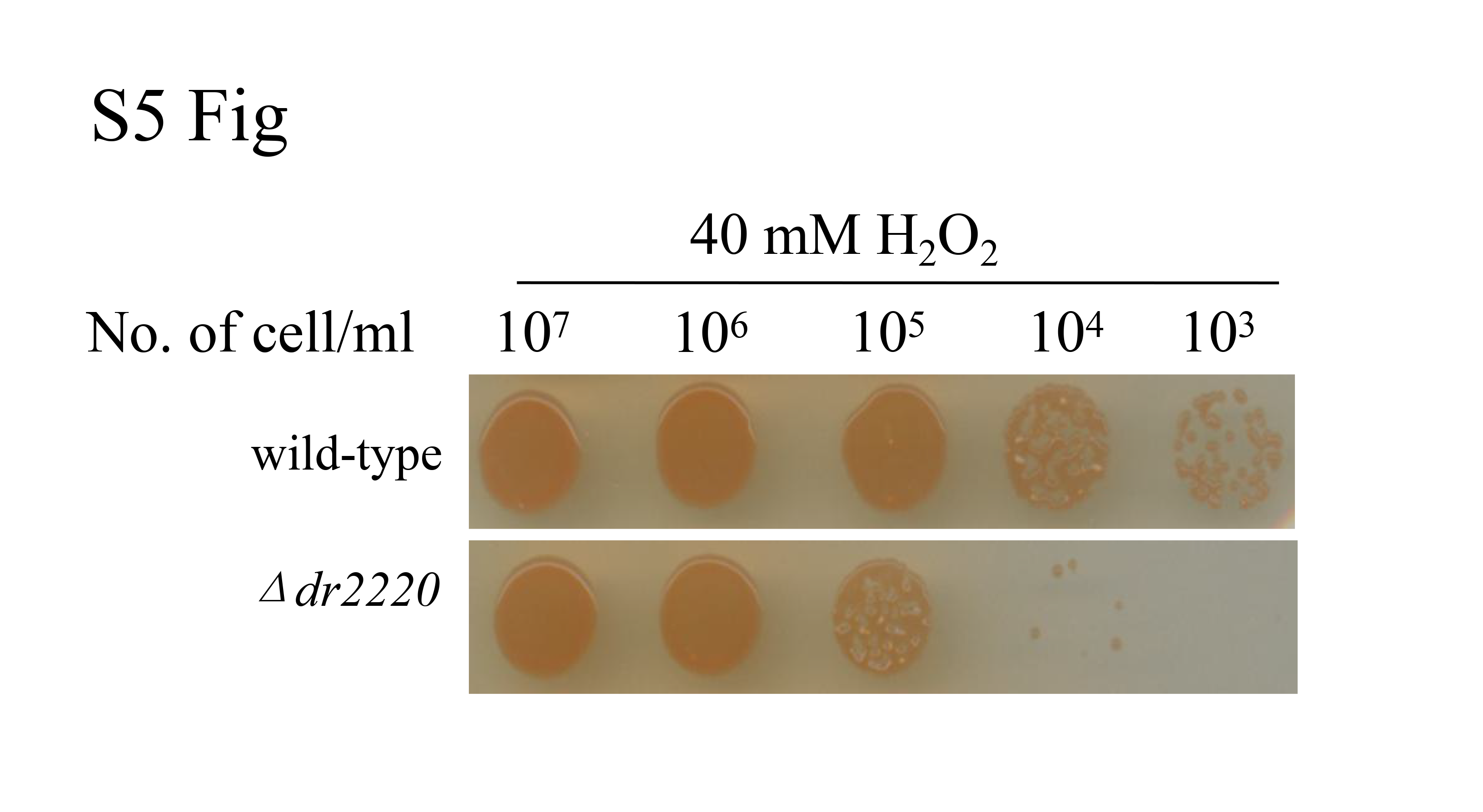

Supplement: S5 Fig — (TIF) [file pone.0155010.s005.tif]

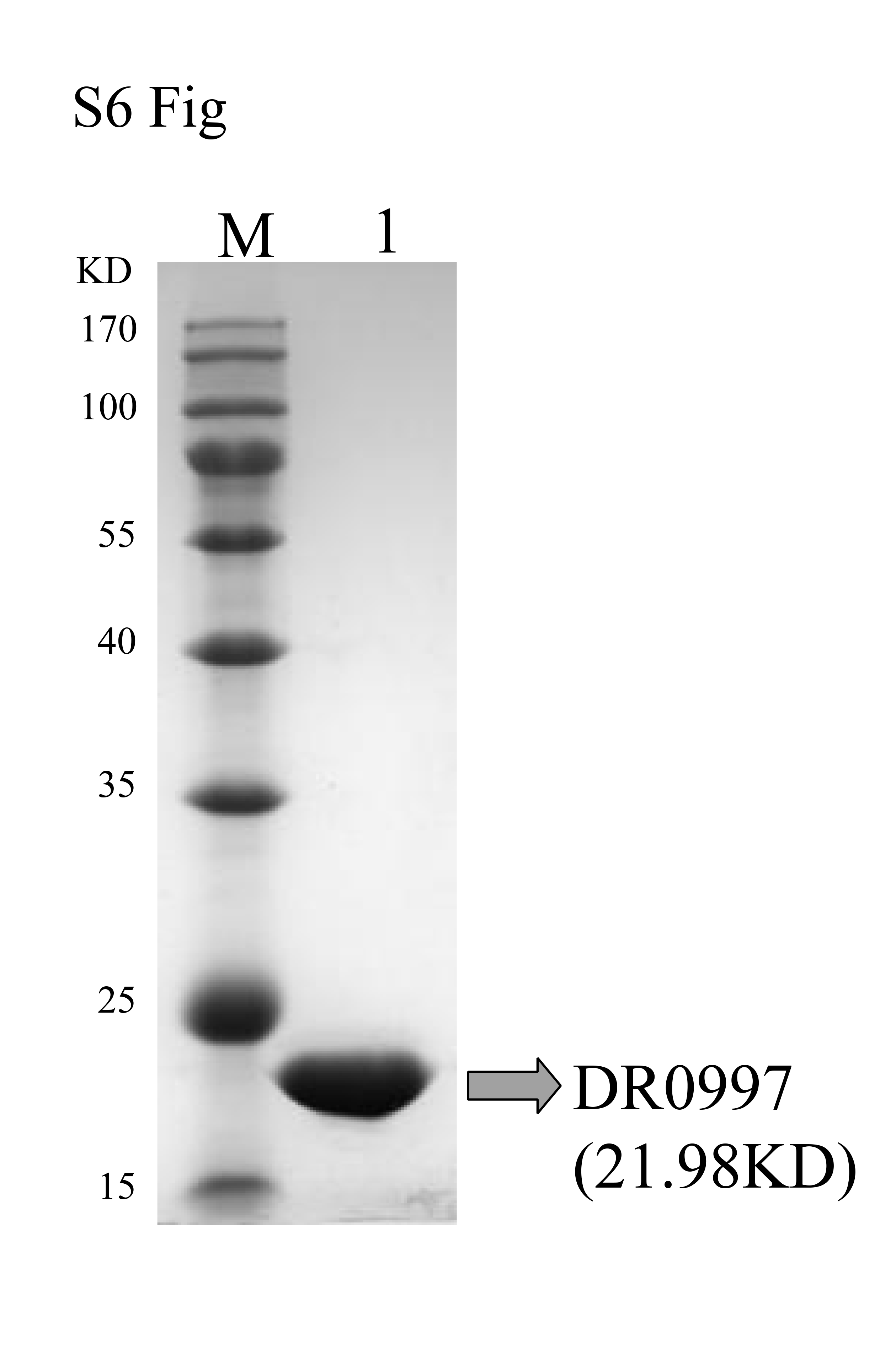

Supplement: S6 Fig — Lane 1, protein ladder; lane 2, DR0997 protein. (TIF) [file pone.0155010.s006.tif]

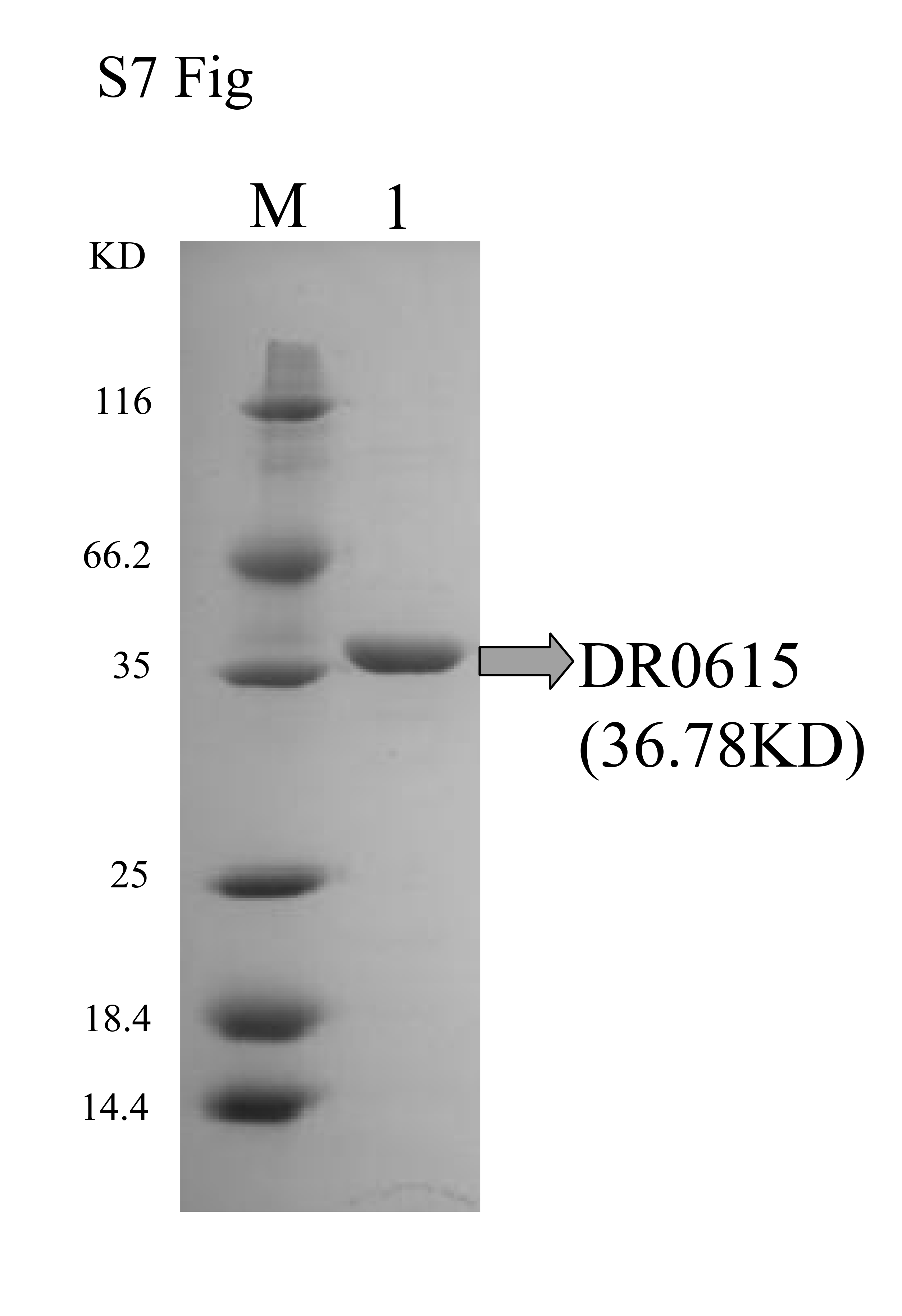

Supplement: S7 Fig — Lane 1, protein ladder; lane 2, DR0615 protein. (TIF) [file pone.0155010.s007.tif]

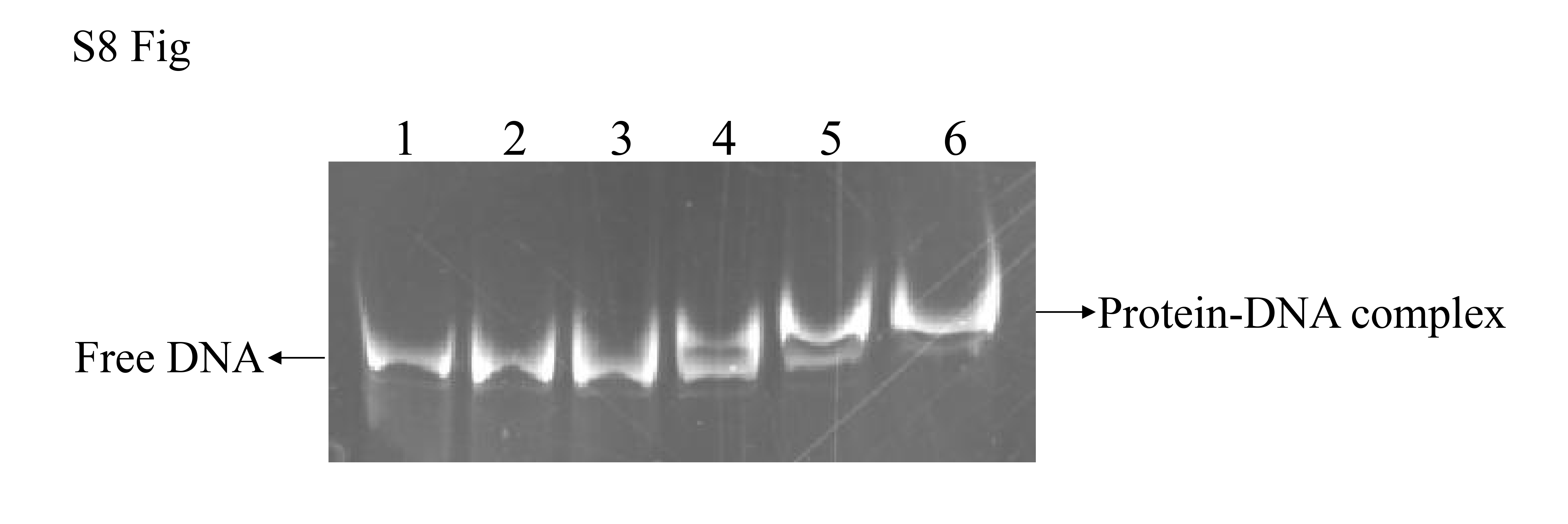

Supplement: S8 Fig — Lane 1, 100 ng of the indicated DNA fragment; lanes 2 to 6, 100 ng of the indicated DNA fragment with increasing concentrations of DR0997 (0.1, 0.25, 0.5, 1, and 10 μM, respectively). (TIF) [file pone.0155010.s008.tif]
