# Supplementary material for: Cyclic AMP Receptor Protein Acts as a Transcription Regulator in Response to Stresses in Deinococcus radiodurans
Source: PLoS One. 2016 May 16;11(5):e0155010. doi: 10.1371/journal.pone.0155010 (PMC4868304; doi:10.1371/journal.pone.0155010)
Supplement: S1 File — (DOC) [file pone.0155010.s009.doc]

**S1 File. Primers used in this study**

**A. Primers for genes knockout**

0997upF: 5′CACCACTCTGACTCGCCGAGAG 3′

0997upR: 5′ATCTAT**GGATCC**CGGAAAAGGTGAAGGGGAG 3′

0997downF: 5′AGAGTC**AAGCTT**TTGCCTGGGCACCCTCTT 3′

0997downR: 5′GCCATGCTGATAAAGCGTACCC 3′

2362upF: 5′GCGGTCTCGTTGTCCCGGTAAAAG 3′

2362upR: 5′AA**GGATCC**GTCTGCCCGGAAGGAT 3′

2362downF: 5′CCG**AAGCTT**GCCCGAGTTGATTGAC 3′

2362downR: 5′GGAATCGGCGTCTTCGAGCGTTT 3′

1646upF: 5′GCTGGCACTGGAATCGGAAGGC 3′

1646upR: 5′ATAT**GGATCC**GGGGTCGTCGGGGTGAAAGAT 3′

1646downF: 5′ATAT**AAGCTT**CTACCGCCGCATCGTGCTTACC 3′

1646downR: 5′CGTTGAGTCGAGCAGCGTGGT 3′

0834upF: 5′CACGGCGTTTACCCTCAGCATCC 3′

0834upR: 5′TA**GGATCC**AGGAAGCCTCCAGAAGAAAG 3′

0834downF: 5′GCT**AAGCTT**GAGAAGCGGGGAAGACAG 3′

0834downR: 5′CTGACCCAGTCGCTCCTCAAGCAC 3′

Comdr0997F: 5′GGAATTC**CATATG**ACCCAGACCCAGACTGCTG 3′

Com0997R: 5′TTGC**GGATCC**TCATCTTGACCACAAATCGGCG 3′

**B. Primers for protein expression plasmids**

PHMTdr0997F: GGAATTC**CATATG**ACCCAGACCCAGACTGCTG

PHMTdr0997R: TTGC**GGATCC**TCATCTTGACCACAAATCGGCG

**B. Primers for QRT-PCR analysis**

RT-0997F: GGGCTGACCCTGCGCTATGTC

RT-0997R: TTGCCGACGAGGCGGTAGATG

RT-1646F: GGGCTGGTCTTTCTGGAAGTCTC

RT-1646R: GAAATCGCCGAGCAGTTTGGTG

RT-2362F: TCAGCACCAAGCCCGACCTC

RT-2362R: CGGGCGATGTCTTCCTGGTT

RT-0834F: ACCGTGCGTCACATCCAGCC

RT-0834R: GTACGTGAATGCCGCCCTGC

RT-1998F: GGGCGTGGACAAGCGTATTC

RT-1998R: GTAGACGGGGGCTTCCTGCT

RT-a0259F: GGGCAAGGCTCACCTCGTCA

RT-a0259R: CGCCCTGGTCAATGGTCTCC

RT-a0146F: GGCGCCGAGTACCAGCTGC

RT-a0146R: CGTGCCAGGGGTTGAACTCCAG

RT-1546F: TGTCGCTTTCCGCCTGTGC

RT-1546R: TCGGGTTCTCGTGGATGTGC

RT-a0202F: GAGCCGCAACCACGATACGC

RT-a0202R: GTCTTGTAGTCGTCGGGGTTGG

RT-1279F: GGCAAGCTCGATGTCGTGTCC

RT-1279R: GGCGGCGGTTCTGGTAGTTG

RT-1771F: CATCATCCCCTGGACCAAAAAG

RT-1771R: GCCGGTAGACAACCTCAAAAGC

RT-2275F: CCATTCCGCTTACCTTCCTCCT

RT-2275R: ACCTCAGCCCACTTTCCAGTCC

RT-1354F: ACCTCATCAAGCAGCACCGC

RT-1354R: CAGGCCCGAATTTTTACGCA

RT-1274F: GCGTGAAGTGCGTGAGGGA

RT-1274R: GGTGGTCAGCGTCGGGAAAG

RT-0596F: TGGACGACCGCGACAAGAAGT

RT-0596R: TGATGAAGCCGAGCTGAATCAG

RT-0440F: CTGGGACTGGTCGAAGGGGAT

RT-0440R: TGGGGCCGTAGGCGTGAAT

RT-0040F: TAGGTGGCGTGCGTGTGGAG

RT-0040R: GCAGCGAGGTGATGAGGGTG

RT-1477F: ACACCCCCAGCGTCGTCTTT

RT-1477R: GAGCATCCGGGCGATTTCTT

RT-1916F: ACGTGGAGGAAGGCCAGAAGG

RT-1916R: CTCGACCCAGGGTTGGTTGAA

RT-2340F: GGACTCGGTGGCTGCTCTGAC

RT-2340R: TTTTCTCGCGAACCTGGTTGA

RT-A0346F: CCCCTGGCCTTTTTAATCTGTT

RT-A0346R: GAATTTCGATGTCGCCGTCGTC

RT-2606F: GCTGGTGGTGCTGGAAGAGGG

RT-2606R: AGGTCGTGGCTGATGGGGTAG

RT-2418F: CGAAGGCAGCAACGTGGTG

RT-2418R: CGGAGGCAAAGAAGGGGAGG

RT-0167F: CCTTGCCCCTCTGGGGTAAG

RT-0167R: TCAGGCTGTGGGTGTCGCGT

RT-0003F: GTCAAAAGATGAAAAAGCCCC

RT-0003R: CTCACCCTCCACGAACAGGTA

RT-0070F: GAACGTGGAGCATGAAAGCC

RT-0070R: TCTCGATGGGAAACTGGTAGC

RT-1440F: CCGCGCCATGACCTTTATG

RT-1440R: GTGTTCGCTGTGGGCTTCC

RT-2574F: TCGTGAATTGCGCAGCGAA

RT-2574R: GGCGAGGGTCTGGAGGGTT

RT-0326F: CGCCCACCTCGACCTCTTTC

RT-0326R: GCGTCAATCGTTTCGCCCTT

RT-1126F: GTGGGCGGTGTACGAGGAA

RT-1126R: CGGACCAGAGCACGGTTTT

RT-1819F: AACGACGAGCGGGCATACA

RT-1819R: CTCGGAAAATCGGCAATCAG

RT-1089F: GACCCCGTTTTGCTCCTCG

RT-1089R: CCTCCGCTTGCATCTCCTCAT

RT-0819F: AACAGGCGGTGCTGGAGGG

RT-0819R: AGCGAGGCGGCGAAGAGGT

RT-0198F: GGCGGCTCGCTGGAATACAC

RT-0198R: TCTTCGTCACCGGGACGTTG

RT-2220F: GCAGTTCATCACCAGCAGCG

RT-2220R: GCATCAGCTCGATTTTCCCG

RT-1506F: AAGGCTGGAGTCAGGAGGGA

RT-1506R: GCATGGCGACGAGGTAAAAG

RT-0990F: AGCAAGCTGACGGTGGAAGAAA

RT-0990R: TGCCGAGGATGGTGGTAAAGG

RT-0998F: CCACGGGGTACGCTTTATC

RT-0998R: ACGCCGAGTCTTTCAGAAAC

RT-1736F: GGAGCACAGGTGCAGGAGTG

RT-1736R: CGAAGTTGTAGGTGGGGAAGG

RT-1974F: CCCACAAGGACGGCGAAAC

RT-1974R: GCCCACGAGGCACAGGATG

RT-A0006F: GCATCGCATGGCGTTCACT

RT- A0006R: TACCAGGTCTCCGCGTTTGT

RT-1929F: ACGGCTACGCCATCAACCC

RT-1929R: CGACCAGACCGCCCACAATC

RT-1689F: CCCCGAACTCAAGGAGCAAC

RT-1689R: CATCAGGAACCCGAAGGCAA

RT-2531F: CGCTCGATAAAAAAGCCGGG

RT-2531R: AGGTTGGGGGTCGCACTCAC

RT-0349F: GCCTCGGTTATCCGCACTTAC

RT-0349R: AGACCAGAATCGGGCCTTTGT

RT-1343F: GCCTACACCGAAGACCCCAT

RT-1343R: GAACTTGACGAGGTTGCCCAT

**C. Primers for promoter regions**

P*dra0006* F: 5'GGTGATCATGCCCTCAGAGTGC3'

P*dra0006* R: 5'TGTTCCAGTTCGTCATGGTCCG3'

P*dr2531* F: 5'TTGCTGATCGAAATACGACTGAATG3'

P*dr2531* R: 5'CGGCGAACTGTGAAAAGATACCCT3'

P*dr0997* F: 5'GGTGAAACCCTCCAAATCAGCCT 3'

P*dr0997* R: 5'TAGGTCACGGTGTCCACGAAGGT3'

P*dr1646* F: 5'CCACGCCGTCGGCAGTCAC3'

P*dr1646* R: 5'GAGCGGCTGGCTGTTCGAGTC3'

P*dr0834* F: 5'GTCCTGGGCTGACCCGTAACC3'

P*dr0834* R: 5'GGGACATAGGAAGCCTCCAGAAG3'

P*dr2362* F: 5'CGCCCCATTTCCCTGCCT3'

P*dr2362* R: 5'CCTCACGAGTCGGACGTTTCTAG3'

P*dr1819* F: 5'CTCAGAGTAAGCCCCCGCACAG3'

P*dr1819* R: 5'GTCAGGCACACCAGCCCGAG3'

P*dr1736* F: 5'GTGCGAACGCTTTTTATTGTTTGTC3'

P*dr1736* R: 5'CCACGGAGCCAGTCAGCAAAAG3'

P*dr1477* F: 5'TGCGGCTTACCCCAGGTCATC3'

P*dr1477* R: 5'GGGAGGATTTTACGCCCTGAAC3'

P*dr1689* F: 5'GCACCTGCGGGCGTTTCTG3'

P*dr1689* R: 5'CACTGCACTTTCCCGTCAGACTG3'

P*dr1929* F: 5'TTCTGGGCGTCTTCATGACCTACTT3'

P*dr1929* R: 5'CCGTAAATTTCATTGTGCTCACCTC3'

P*dr0998* F: 5'CTCAGCACTTTGCCCCTCACAG3'

P*dr0998* R: 5'ATCACGGTGCAGTTCGGGGTC3'

P*dr1974* F: 5'GGACTCAAAAAGTCTGCATAAAGCG3'

P*dr1974* R: 5'CCCCACGTCCACGTTCATGGT3'

P*dr0349* F: 5'TTAGGCATTTGTCTATGGGGCACT3'

P*dr0349* R: 5'GGCCTGGAGCCCAAAAAGATGT3'

P*dr0990* F: 5'GGCGGTGATCTTCATGACAGC3'

P*dr0990* R: 5'ACATTCTCCCCTGCCCTCTCTAC3'

P*dra0346* F: 5'CACTGTCATCTGGACGTTTCTTTC3'

P*dra0346* R: 5'TTGCTTTAGCCCTTGCCATACTG3'

P*dr2220* F: 5'GGACATCCTGACCCGTGCG3'

P*dr2220* R: 5'CATTTTGGTGCCGTCCTCCG3'

P*dr1921* F: 5'GACCGGGTTGCAGGGTGACG3'

P*dr1921* R: 5'CGGGCTCGTTTTCCAGTATCTCC3'

P*dr1506* F: 5'CGCCTTCGTGATGTTGCTGACC3'

P*dr1506* R: 5'GTGAAAAGAGGCACGAACGACT 3'

P*dra0006S1* F: 5'GGTGATCATGCCCTCAGAGTGC 3'

P*dra0006S1* R: 5'TGAAGTCTCATAAGGGGACGTGACT 3'

P*dra0006S2* F: 5'CGTCCCCTTATGAGACTTCACACAC3'

P*dra0006S2* R: 5'TGTTCCAGTTCGTCATGGTCCG3'

P*dr2531S1* F: 5'TTGCTGACCGCCTCACCGAT3'

P*dr2531S1* R: 5'CCGGAGGACGTATGAAGGACATC3'

P*dr2531S2* F: 5'CGGTGAAGCTGAGCAGACAACG3'

P*dr2531S2* R: 5'ATGTCCAGCAGCCTACTCCCT3'

P*dr1819S1* F: 5' ATCGGAACCTCCTCAGAGTAAGCC3'

P*dr1819S1* R: 5' GTCGCGCTCCCATTTCTTACG3'

P*dr1819S2* F: 5'GTAAGAAATGGGAGCGCGACTG3'

P*dr1819S2* R: 5'TCAGGCACACCAGCCCGAG 3'

P*dr1506S1* F: 5'GCCTTCGTGATGTTGCTGACC3'

P*dr1506S1* R: 5'GGCAAAAGTGAGGACAGCAAAAC3'

P*dr1506S2* F: 5'TTCTGAGTCCTACCTTCTCCGCC 3'

P*dr1506S2* R: 5'CGTCATCCAGTCACTTACGAGCAT 3'

P*dr1477S1* F: 5'CTTACCCCAGGTCATCGGCG3'

P*dr1477S1* R: 5'GAGAAAGGGGCGGAGAGAAGACT3'

P*dr1477S2* F: 5'AACACGACTTTTCAGTGGGCG3'

P*dr1477S2* R: 5'GGTCACGGGGAGGATTTTACG3'

P*dr1929S1* F: 5'GCGTTCGTCTCGGTGCTGTTT3'

P*dr1929S1* R: 5'CTATTTTTCAGACGGACAGTCACAG3'

P*dr1929S2* F: 5' GCGTTCGTCTCGGTGCTGTTTT3'

P*dr1929S2* R: 5' CTATTTTTCAGACGGACAGTCACAG3'

P*dr0998S1* F: 5'CAGCACTTTGCCCCTCACAG3'

P*dr0998S1* R: 5'CAGGGCGTCGTTCATATAGGC3'

P*dr0998S2* F: 5'GTGGTCAAGATGACGCCTATATG3'

P*dr0998S2* R: 5'CTCGGCCCGTTCGATCATG3'

P*dr1689S1* F: 5'CCTGCGGGCGTTTCTGGAG3'

P*dr1689S1* R: 5'CAACTCCTGACGAGCGGGAATAC 3'

P*dr1689S2* F: 5'GTCTTTTGTATTCCCGCTCGTC3'

P*dr1689S2* R: 5'AGCAAGAAAGGAAGAGGGGCAC3'

P*dr1736S1* F: 5'TGCGAACGCTTTTTATTGTTTGTC3'

P*dr1736S1* R: 5'GAGTGCCTTGCAGGGCGTGAAC3'

P*dr1736S2* F: 5'GGAAACTCCGAACGGCGTGAC3'

P*dr1736S2* R: 5'GACACGCCAAGTGGGCACAGAA3'

P*dr1736S3* F: 5'ATTAATGGTTCTGTGCCCACTTGG3'

P*dr1736S3* R: 5'CAGCCACGGAGCCAGTCAGC3'

P*dr1974S1* F: 5'CATCCCCTTTAATCTTTCCGCC3'

P*dr1974S1* R: 5'AATCACGATATTTCTCAGCGCAAC3'

P*dr1974S2* F: 5'GGTTTTCAAAGAGCAGGCAAGG3'

P*dr1974S2* R: 5'GTTCATGGTGACGCCGGGC3'
