# Supplementary material for: Cyclic AMP Receptor Protein Acts as a Transcription Regulator in Response to Stresses in Deinococcus radiodurans
Source: PLoS One. 2016 May 16;11(5):e0155010. doi: 10.1371/journal.pone.0155010 (PMC4868304; doi:10.1371/journal.pone.0155010)
Supplement: S2 File — (DOC) [file pone.0155010.s010.doc]

**S2 File. CRP binding sites**

***dr_A0006* cyclase / dehydrase**

(-101) CRP site Ⅰ

AGGGCGTAGTGCCAGCTGTCAGGCGACCCGGATGACATT**TGTCA**GCCCCG**TCATT**TGTCAGGGCAGTCA

CRP site Ⅱ

CGTCCCCTTA**TGAGA**CTTCAC**ACACA**GTGACACAGTGATCTATTGGACCGGCGGTCATCCCCTCAACTG

CGGACCATGACGAACTGGAACAGGGAGCAGCCC (+60)

***dr_2531* thiosulfate sulfurtransferase**

CRP site Ⅰ

GCGACGGCTTGCTGATCGAAATACGACTG**AATGA**TGTCCT**TCATA**CGTCCTCC**GGTGA**AGCTGA**GCAGA**

CAACGGACCGGCGAGCAGTCCAGACTGAACAAGGGTAGCCGCTTTTGCCGCCGCGCCAGGGAGTAGGCT

GCTGGACATGACCCCGCCCTCGCCGCT (+20)

***dr_1819* (uvsE)**

*（-120）* CRP site Ⅰ GCGGGTGGCAAAC**CGTGA**GAAATCT**TCCCC**TGCAAAGAGGCCCGGCGGCGGGCGGCCCCCCAGCGAGCG

GCCCAGCAAAATCTGCGCGCGCTGCGGCCTGCCCTTTTCTTGGCGTAAGAAATGGGAGCGCGACTGGGA

CAACGTGCGCTACTGCTCGGAG （+40）

***dr_1506* (****NADH-quinone oxidoreductase subunit A)**

*（-164）* CRP site Ⅰ

TTCGCCTT**CGTGA**TGTTGC**TGACC**CTGTTCGTGGTGGTACGTGACGTGAGCCGCTTTTTCTGAGTCCTA

CCTTCTCCGCCGCTGGCCTGTTCGGGCTGGCGGCGTTTTGCTGTCCTCACTTTTGCCGTCCTCCGGGCG

TCCCGCGCCGCTGTCCGTGCTAGAACATGAGTGTTCTACAAATCAGGCTGACACCAGACAGACTCTCTT

CRP site Ⅱ

TTTTTGCAGTCGTT**CGTGC**CTCTTT**TCACG**AG （+75）

***dr_0990* (AcuB)**

*（-120）* CRP site Ⅰ

CCGGTTTCTGAGCCG**AATGA**AGCACG**TCACA**GTTGTCTCACACGTCCGGTCATTCCGCCGTCAG**CTTGA**

CRP site Ⅱ

CCCGGG**CCGCA**GTAGAGAGGGCAGGGGAGAATGTGGCGCTAAACTCCATTTATGCTCGTAAGTGACTGG

ATGACG （+21）

***dr_1477* (recN)**

*（-218）* CRP site Ⅰ

ATAGGCACCAA**CGTGC**AGCAGT**ACACC**GTCAAGGGAAACACCCTGACCGTCCCGCG

CGTGGACCGTTGCGGCAAGTTCAACACGACTTTTCAGTGGGCGACAGTTCCCCTCCCG

AAGCCGACCCAGACGGGCTTTTAGTCTTCTCTCCGCCCCTTTCTCCTCCCGAC**TGTGC**

CRP site Ⅱ

CCTGAC**TCCCG**CCCCCGCAGTAAGTTCAGGGCGTAAAATCCTCCCCGTGACCCGCAA

GG （+13）

***dr_1929* (glycerol uptake facilitator protein)**

*（-100）*

ACATCGTGGCTGGGCAGGCGACCCAGTTTCCCCTGTGACTGGTTTCCCCTGTGACTGTCCGTCTGAAAA

CRP site Ⅰ

ATAGGTAAACTTTGTCACAAAAAAAGGAGAGGTGAGCACAATGAAATTTACGGCA （+24）

***dr_0998* (ATP-binding protein involved in chromosome partitioning)**

*（-110）* CRP site Ⅰ

TCC**CGTGC**CGGGCT**TCTCT**TGCCTGGGCACCCTCTTTTTTGGCGACGGCGCGGCGCTCTGTGAGTTTCG

TCGTTGCCAGAACGCCGATTTGTGGTCAAGATGACGCCTATATGAACGACGCCCTGCTGCGTGCCCTGA

CRP site Ⅱ

GCAC**TGTGA**ACGACC**CCGAA**CTGCACCGTGAT （+60）

***dr_0349* (ATP-dependent Lon protease)**

*（-240）*  CRP site Ⅰ

CGGGGTGGCCCGGCTCCCTCAACTTCCGCTCAAGTTTC**TGTGA**GCTTCG**TGATA**GTGAATTTGCCGTAC

TGTACGCGCTCTAAGCGTGTTGGCTTACCCTGGTAAGCACTTGCCTGAATGACGAAAACTTGATATATT

TGCACTCAGGTTTTTTGGTAAGACATCTTTTTGGGCTCCAGGCCCCCTTGCCAAGCCACCCCTTCGCGA

CGGCCCCCTGCGGGTCAAAAGGAGCGTCAACGGATGCCCG （+7）

***dr_1689* (glucose-1-phosphate adenylyltransferase)**

*（-180）* CRP site Ⅰ

CCT**TGCGG**AAAAGT**TCAGA**CTTGGTTTGCCGACTTACAAGCTAAGTCAAAAAAGTAGCGCTGGGACGCG

CRP site Ⅱ

TCTTTTGTATTCCCGCTCGTCAGGAGTTGCCTGGAAAGGTTCACCTACTC**GCTGA**TTCCGGG**CCACG**GG

GTCGGCGGTAGAGTGAGGACGACAGTCTGACGGGAAAGTGCAGTGCATTTCGGGGGT （+15）

***dr_1736* (cpdB)**

*（-99）* TTTTTATTGTTTGTCAGCAAGGTTCACGCCCTGCAAGGCACTCTGGGAAACTCCGAAC

GGCGTGACAATCGGTCAGATTATTAATGGTTCTGTGCCCACTTGGCGTGTCATAAAAAT

CRP site

**GTGA**CACGCT**TCTCT**CTTCAGCTTTTGCTGACT （+51）

***dr_1974* (ATP-dependent protease LA)**

*（-120）*

CGCCTGTGACCCTAGACTTCAGGGTACCCGCAGGTGGGGTTTTGACCCTCCCCTGGCGGCGCATCCCCT

CRP site

TTAATCTTTCCGCCCC**TGCGG**CGGTTT**TCAAA**GAGCAGGCAAGGAGCAAGCATGATCTGGGAACTTCCC

GTAGTTGCGCTGAGAAATATCGTGATTCTGCCCGGCGTCACC （+60）

***dr_2220* (terB)**

*（-120）*

CCACCCACACCCGCTGGGCCCAGGACATCCTGACCCGTGCGGAACTTTACGGCGTGAGCCTCGTACCTG

CRP site

AAGCAGAAGCTCTGCATT**TCTGA**CTTCAAT**TCACC**CTCGGGAGGACTCGCAATGGGATTTTTCAACAAG

CTCCGGCAACTGGCGGAGGACGGCACCAAAATGGCGCAGGAC（+60）

***dr_0997*** **(CRP/FNR family transcriptional regulator)**

*（-179）* CRP site

CGGTGAAACCCTCCAAATCAGCCTGC**GGTGA**CATTTCG**TCACC**TCGTAACCTAAATCA

TGATTAAAGAAACAAAACTATTTACAAAATTGTGACCTCGGCCTATACTTTCTGACATC

AAGGCGGACAGATTCGCGCGTCCGCCTCCCCTTCACCTTTTCCGCCTTAGGAGGCGTT

TTCCATGACCC （+7）

***dr_1646* (CRP/FNR family transcriptional regulator)**

*（-179）* CRP site

GGGCTG**GGTGC**GCGACT**GCACG**ATGAATTCAGGGTTGAGGGTGACCACCGTGTGCGGGGTGGGCGGCGA

CTCGAACAGCCAGCCGCTCAGCCGGTCCAGGGCGGCGTCCAGGGTCACGGGGTCGAGCGGCAAGTCGAA

GAGAACCAGACGTTCGGGCAGGCTCTTGAGGCTCATGTAGCGTGATTG （+7）

***dr_2362* (CRP/FNR family transcriptional regulator)**

*（-194）* CRP site

TACCGGCAC**TGTAA**CACCCA**GCACC**CGGCCAGCCTGCGCCCTGACCTAGAAACGTCCGACTCGTGAGGT

CAGGCGACCACTGTCCTCCAAACGGCCCGGCTTAGTCCTCTCATCAAATGGACAAAAAGTAAACCGTCT

TCTGTCGGACAGTCGCCGTTTATCCGCCTGCTTTTCGGTTATCCTTCCGGGCAGACATGAACTA（+8）

***dr_0834* (CRP/FNR family transcriptional regulator)**

*（-158）* CRP site

GCTGACAAAGA**TGTGA**CCCGCC**GCTCA**ACTCGATTGACTGTGGGTGAAGGCGGCGCCTCATGCCCGTGC

CAGTTCTGACAGGGACAATGTAAAAAGTAAAGCGGTCTGCCACGCTCTGTGCCGGTGCCTGCTCCGATT

CTTTCTTCTGGAGGCTTCCTATGTCCCCGACCCCCTCGGCTGCGGCTTTT （+30）

***dr_A0346* (pprA)**

*（-120）*

ACCTGTGCCCTGAACGGCGCCGGGTGCACTTTGGGTGTCACTGTCATCTGGACGTTTCTTTCGC**CGTGA**

CRP site

AAGCTG**GCAAA**GGCACAAAGACCGCCGAAGCTGGGTTTTCCTTATGCCAGAGTGCTACCCCTG （+12）

***dr_1921* (SbcD)**

*（-120）*

GCGGCGGAAGCGGCGGCGCTGAGGCTGAGCAGCAGAGGAAGAACTTTGTTCATGCCCCGATGATGCGCG

Similar CRP site

GCGCGGCTGAAGCATGGC**TGA**CGGCAGG**TCACA**TCGTCGCTTCAGCTTCTAGTGGAGATACT（+11）
