# Supplementary material for: Cyclic AMP Receptor Protein Acts as a Transcription Regulator in Response to Stresses in Deinococcus radiodurans
Source: PLoS One. 2016 May 16;11(5):e0155010. doi: 10.1371/journal.pone.0155010 (PMC4868304; doi:10.1371/journal.pone.0155010)
Supplement: S1 Table — (DOCX) [file pone.0155010.s011.docx]

S1 Table. Statistical analysis of increased multiple transcript of CRP genes after exposure to H_2_O_2_

| Fold change | *dr0997* | *dr1646* | | *dr2362* | *dr0834* |
| --- | --- | --- | --- | --- | --- |
| Increased multiple | 4.16±0.65a | | 1.24±0.29b | 1.91±0.46bc | 2.08±0.35cd |
| LSD | 0.7435 | | | | |

Data were presented as mean value ± standard deviation.

Different letters in the same column indicate significance at the 0.05 probability level.

LSD: least significant difference (p<0.05)

S2 Table. Statistical analysis of survival curves of *D. radiodurans* strains exposure to 50 mM H2O2.

| Strains | 5min(%) | 10min(%) | 20min(%) | 40min(%) |
| --- | --- | --- | --- | --- |
| Wild-type | 0.95±0.03a | 0.57±0.03a | 0.23±0.01a | 0.13±0.01a |
| Δ*dr0997* | 0.65±0.14b | 0.29±0.01b | 0.07±0.01c | 0.01±0.00c |
| Δ*dr0997* Cwt | 0.91±0.03a | 0.54±0.02a | 0.11±0.02b | 0.05±0.01b |

Each column was set as an item to be analysed.

Data were presented as mean value ± standard deviation.

Different letters in the same column indicate significance at the 0.05 probability level.

S3 Table. Statistical analysis of survival curves of Δ*dr2362* and wild-type strains exposure to 50 mM H2O2.

| Strains | 5min(%) | 10min(%) | 20min(%) | 40min(%) |
| --- | --- | --- | --- | --- |
| Wild-type | 0.66±0.11a | 0.40±0.06a | 0.20±0.07a | 0.06±0.02a |
| Δ*dr2362* | 0.59±0.03b | 0.27±0.02b | 0.08±0.02c | 0.02±0.00C |

Each column was set as an item to be analysed.

Data were presented as mean value ± standard deviation.

Different letters in the same column indicate significance at the 0.05 probability level.

S4 Table. Statistical analysis of Catalase activities after 30 mM H2O2 treatment

| Strains | Wild-type | Wild-type  (H_2_O_2_) | Δ0997 | Δ0997 (H_2_O_2_) | Δ0997 Cwt | Δ0997 Cwt (H_2_O_2_) |
| --- | --- | --- | --- | --- | --- | --- |
| Catalase activity | 1663 ± 16b | 2511±25a | 875±18f | 971±21e | 1273±20d | 1454±18c |

Data were presented as mean value ± standard deviation.

Different letters in the same column indicate significance at the 0.05 probability level.
