# Supplementary material for: Cyclic AMP Receptor Protein Acts as a Transcription Regulator in Response to Stresses in Deinococcus radiodurans
Source: PLoS One. 2016 May 16;11(5):e0155010. doi: 10.1371/journal.pone.0155010 (PMC4868304; doi:10.1371/journal.pone.0155010)
Supplement: S2 Table — (DOCX) [file pone.0155010.s012.docx]

S2 Table. Statistical analysis of survival curves of *D. radiodurans* strains exposure to 50 mM H2O2.

| Strains | 5min(%) | 10min(%) | 20min(%) | 40min(%) |
| --- | --- | --- | --- | --- |
| Wild-type | 0.95±0.03a | 0.57±0.03a | 0.23±0.01a | 0.13±0.01a |
| Δ*dr0997* | 0.65±0.14b | 0.29±0.01b | 0.07±0.01c | 0.01±0.00c |
| Δ*dr0997* Cwt | 0.91±0.03a | 0.54±0.02a | 0.11±0.02b | 0.05±0.01b |

Each column was set as an item to be analysed.

Data were presented as mean value ± standard deviation.

Different letters in the same column indicate significance at the 0.05 probability level.
