# Supplementary material for: Cyclic AMP Receptor Protein Acts as a Transcription Regulator in Response to Stresses in Deinococcus radiodurans
Source: PLoS One. 2016 May 16;11(5):e0155010. doi: 10.1371/journal.pone.0155010 (PMC4868304; doi:10.1371/journal.pone.0155010)
Supplement: S3 Table — (DOCX) [file pone.0155010.s013.docx]

S3 Table. Statistical analysis of survival curves of Δ*dr2362* and wild-type strains exposure to 50 mM H2O2.

| Strains | 5min(%) | 10min(%) | 20min(%) | 40min(%) |
| --- | --- | --- | --- | --- |
| Wild-type | 0.66±0.11a | 0.40±0.06a | 0.20±0.07a | 0.06±0.02a |
| Δ*dr2362* | 0.59±0.03b | 0.27±0.02b | 0.08±0.02c | 0.02±0.00C |

Each column was set as an item to be analysed.

Data were presented as mean value ± standard deviation.

Different letters in the same column indicate significance at the 0.05 probability level.
