# Supplementary material for: Cyclic AMP Receptor Protein Acts as a Transcription Regulator in Response to Stresses in Deinococcus radiodurans
Source: PLoS One. 2016 May 16;11(5):e0155010. doi: 10.1371/journal.pone.0155010 (PMC4868304; doi:10.1371/journal.pone.0155010)
Supplement: S4 Table — (DOCX) [file pone.0155010.s014.docx]

S4 Table. Statistical analysis of Catalase activities after 30 mM H2O2 treatment

| Strains | Wild-type | Wild-type  (H_2_O_2_) | Δ0997 | Δ0997 (H_2_O_2_) | Δ0997 Cwt | Δ0997 Cwt (H_2_O_2_) |
| --- | --- | --- | --- | --- | --- | --- |
| Catalase activity | 1663 ± 16b | 2511±25a | 875±18f | 971±21e | 1273±20d | 1454±18c |

Data were presented as mean value ± standard deviation.

Different letters in the same column indicate significance at the 0.05 probability level.
